# Profiling the immune epigenome across global cattle breeds

**DOI:** 10.1186/s13059-023-02964-3

**Published:** 2023-05-22

**Authors:** Jessica Powell, Andrea Talenti, Andressa Fisch, Johanneke D. Hemmink, Edith Paxton, Philip Toye, Isabel Santos, Beatriz R. Ferreira, Tim K. Connelley, Liam J. Morrison, James G. D. Prendergast

**Affiliations:** 1 The Roslin Institute and Royal (Dick) School of Veterinary Studies, University of Edinburgh, Easter Bush Campus, Edinburgh, EH25 9RG UK; 2 Ribeirão Preto College of Nursing, University of Sao Paulo, Ribeirão Preto, Brazil; 3 Centre for Tropical Livestock Genetics and Health, Roslin Institute, University of Edinburgh, Easter Bush Campus, Edinburgh, EH25 9RG UK; 4 The International Livestock Research Institute, PO Box 30709, Nairobi, 00100 Kenya; 5 Centre for Tropical Livestock Genetics and Health, ILRI Kenya, PO Box 30709, Nairobi, 00100 Kenya

**Keywords:** Cattle, Subspecies, DNA methylation, Chromatin, Expression, Immune cells

## Abstract

**Background:**

Understanding the variation between well and poorly adapted cattle breeds to local environments and pathogens is essential for breeding cattle with improved climate and disease-resistant phenotypes. Although considerable progress has been made towards identifying genetic differences between breeds, variation at the epigenetic and chromatin levels remains poorly characterized. Here, we generate, sequence and analyse over 150 libraries at base-pair resolution to explore the dynamics of DNA methylation and chromatin accessibility of the bovine immune system across three distinct cattle lineages.

**Results:**

We find extensive epigenetic divergence between the taurine and indicine cattle breeds across immune cell types, which is linked to the levels of local DNA sequence divergence between the two cattle sub-species. The unique cell type profiles enable the deconvolution of complex cellular mixtures using digital cytometry approaches. Finally, we show distinct sub-categories of CpG islands based on their chromatin and methylation profiles that discriminate between classes of distal and gene proximal islands linked to discrete transcriptional states.

**Conclusions:**

Our study provides a comprehensive resource of DNA methylation, chromatin accessibility and RNA expression profiles of three diverse cattle populations. The findings have important implications, from understanding how genetic editing across breeds, and consequently regulatory backgrounds, may have distinct impacts to designing effective cattle epigenome-wide association studies in non-European breeds.

**Supplementary Information:**

The online version contains supplementary material available at 10.1186/s13059-023-02964-3.

## Background

Globally, almost 1.3 billion people depend on livestock for their livelihood, with cattle providing a significant source of nutrition to over 6 billion people [1]. However, infectious diseases are a major global constraint to cattle production, with many diseases being zoonotic and consequently also of direct relevance to human health. In recent years, substantial global investment has managed to disentangle the genetic basis of a large number of complex traits in cattle. However, a large amount of the variation underpinning important phenotypes between animals remains unexplained. There is consequently an increasing focus on the potential relevance of non-genetic variation, including gene expression, DNA methylation and chromatin, to important cattle phenotypes. While reference resources and tools for understanding and exploiting such ‘beyond-genome’ variation are available for human and laboratory organisms, even baseline knowledge is lacking for most cattle breeds—despite the clear importance and potential translatability of heritable traits in this organism. To fully understand the non-genetic component of cattle traits, the importance of gene by environment interactions, and how genetic variation may influence phenotypes via the methylome, it is necessary to profile chromatin and methylation states across the genome and to understand how and where it varies across animals and breeds.

Cattle are unusual in that they likely exhibit unusually high levels of regulatory divergence due to having been domesticated at least twice. The primary taurine (*Bos taurus taurus*) and indicine (*Bos taurus indicus*) cattle lineages are estimated to last have had a common ancestor over 210,000 years ago [2–4]. These lineages can be further subdivided, with, for example, the taurine cattle migrating both North to Europe and West to Africa, leading to the isolation of these sub-lineages for several thousand years [5–8]. Admixture events and selection pressures imposed by the environment and local production systems has led to further genetic and phenotypic divergence between breeds, but the corresponding extent of underlying epigenetic divergence remains largely unknown. In the few instances where this has been analysed between cattle breeds, differential methylation analysis has identified a number of candidate genes that potentially contribute to key phenotypic differences. For example, a comparison of DNA methylation and gene expression profiles of longissimus dorsi muscles between Japanese black and Chinese Red Steppes cattle identified genes that might contribute to differences in meat quality [9]. While a comparison of the methylomes of two Creole cattle breeds living in tropical environments with three Iberian breeds identified candidate genes that may be important in tropical adaptation processes such as the immune response, nervous system and heat resistance [10]. These studies suggest that environmental changes can have a measurable impact upon methylation patterns.

Even less studied across cattle populations is the landscape of chromatin accessibility. Techniques to map chromatin accessibility, such as DNase I hypersensitive sites sequencing (DNase-seq) and assay for transposase-accessible chromatin using sequencing (ATAC-seq), have been used extensively to profile functional genomic elements in humans [11] and classical model organisms [12–14]. These data have shed light on the mechanisms governing a wide variety of biological processes, including disease [15–17] and cellular differentiation [18, 19]. In contrast, chromatin accessibility maps measured using ATAC-seq are available for considerably fewer somatic tissues and cell types in cattle, with the overwhelming majority from European taurine cattle breeds [20–25].

Epigenetics has been shown to play a crucial role in the development and differentiation of the human immune system, as well as in related pathologies [26–28]. While studies are limited, there is also evidence to suggest that epigenetics plays a similarly important role in the cattle immune system and thus may contribute to a breed’s susceptibility to a given disease. For example, DNA methylation has been implicated in the regulation of the immune response to mastitis [29, 30], lipopolysaccharide [31] and *Mycobacterium bovis* [32]. These studies have started to characterise epigenetic signatures related to infection, yet the degree of natural epigenetic variation in the immune system between cattle populations is mostly unexplored.

Further studies are required to characterise how variation in the methylome and spectrum of chromatin accessibility shape cattle phenotypes. A common approach in human studies is epigenome-wide association studies (EWAS), which correlate methylation differences between individuals to their phenotypes. However, disentangling the effect of potential confounding factors, such as cell type heterogeneity, is a significant problem in EWAS. Blood is the most commonly studied tissue in EWAS due to its accessibility across large numbers of animals. However, blood is composed of a number of individual cell types. Within such heterogeneous populations, each cell type has a unique epigenetic profile, and thus changes observed between samples may merely reflect a change in cell type proportions between them, rather than a change at the epigenetic level [33]. To account for this a number of cellular deconvolution approaches have been developed, which estimate the cell type proportions in each sample in silico allowing for them to be accounted for in downstream analyses. The majority of these approaches are reference-based, i.e. they require a reference panel of methylation states for each cell type that can be used to deconvolute composition. However, there are currently no suitable reference panels available that would enable cell type deconvolution and accurate EWAS across different cattle breeds.

In this study, we consequently generated multi-layered, genome-wide omics maps of genetic variants, gene expression, DNA methylation and chromatin accessibility covering seven major blood immune cell types across three genetically diverse cattle groups: Holstein Friesian (European taurine), N’Dama (African taurine) and Nelore (indicine). These breeds are representative of the major cattle lineages and were used to explore the degree of natural epigenetic variation between animals and to identify breed- and cell type-specific epigenetic signatures. Furthermore, to enable cattle EWAS from whole blood samples, we applied the deconvolution algorithm, CIBERSORTx, to compute the cell type compositions of complex cellular mixtures based on their DNA methylation profile. The data generated in this study will consequently provide a foundational resource for interpreting cattle genomics studies, such as the functional validation of Genome-Wide Association Study (GWAS) variants, as well as facilitating EWAS studies that can account for potential confounding by cell type.

## Results

### Generation and validation of the chromatin accessibility, DNA methylation and transcriptomic immune profiles

**Fig. 1:**
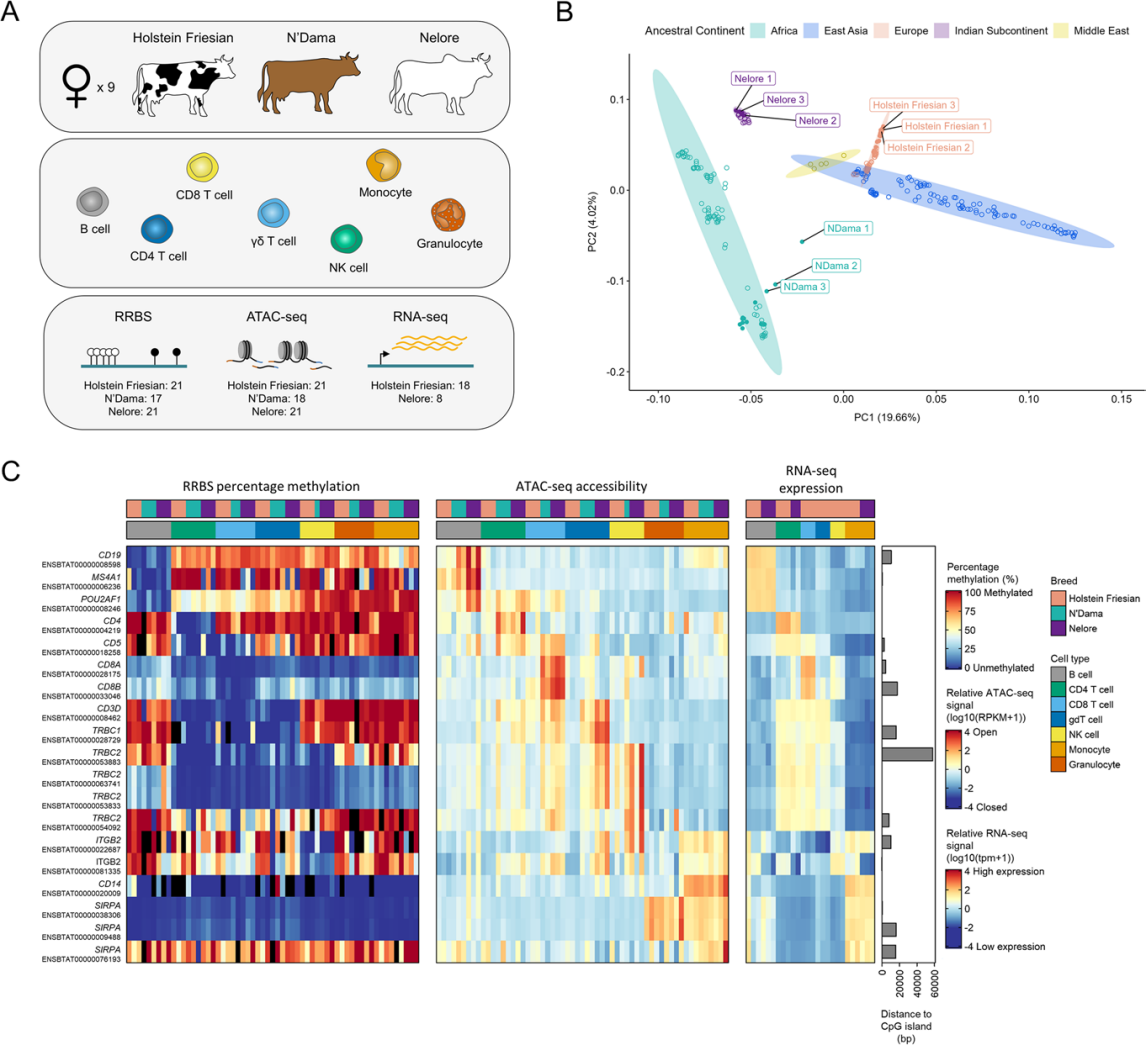
Investigation of chromatin landscapes in primary blood cells from three genetically diverse cattle breeds. **A** Overview of cell types collected from up to nine female Holstein Friesian, N’Dama and Nelore cattle (three of each breed) for RRBS, ATAC-seq and RNA-seq. The total numbers of RRBS, ATAC-seq and RNA-seq samples collected for each breed is shown at the bottom of the schematic. **B** PCA of cattle genotype data. Analysis was performed using 27,379,808 variants from 298 cattle. Filled points symbolise Holstein Friesian, N’Dama and Nelore cattle, of which the labelled points highlight the animals used in this study. **C** Percentage methylation, chromatin accessibility and gene expression of immune-related genes. Heatmaps showing the percentage methylation (left) and chromatin accessibility (middle) at promoters (defined as 1000 bp upstream and 500 bp downstream of TSSs) of immune-related genes and their corresponding expression (right). Bars on the right show the distance of the TSS of each transcript to the nearest CGI. Black bars in the methylation heatmap represent samples where fewer than 5 CpG sites covered by a minimum of 5 reads were found at a given promoter. SIRPA denotes SIRPα and gdT cell denotes γδ T cell. This figure also highlights that, consistent with previous reports, the number of CD4+CD8+ double positive T cells is likely small given the expression of CD4 and CD8 are largely specific to the respective cell types [34]

Genome-wide DNA methylation and chromatin accessibility were profiled using Reduced Representation Bisulfite Sequencing (RRBS) and ATAC-seq, respectively, targeting seven key blood immune cell types (B cells, CD4 αβ T cells, CD8 αβ T cells, γδ T cells, NK cells, monocytes and granulocytes) from Holstein Friesian, N’Dama and Nelore cattle (Fig. 1A). Due to the challenges of sample collection and cell isolation for the N’Dama breed, four ATAC-seq and three RRBS samples for this breed were excluded from the study. Consequently, in total, 119 libraries were generated across the three breeds, with data for B cells, CD4 αβ T cells, γδ T cells and monocytes across all nine cattle (Additional file 2: Table S1 and Table S2). For comparison in downstream analyses, 26 RNA sequencing (RNA-seq) libraries were also generated for a subset of cell types from the Holstein Friesian and Nelore cattle (Additional file 2: Table S3).

To verify that the sampled animals were genetically representative of divergent cattle, each was whole genome sequenced and the genotypes compared by principal component analysis (PCA) with data from a previous study for an additional 289 cattle [35]. The genetic relationship between cattle breeds was found to be largely reflective of their ancestral geographical distribution (Fig. 1B). The positioning of N’Dama 1 suggests that this animal may be the offspring of a relatively recent cross between an N’Dama and a European taurine animal. Nevertheless, the three groups of sampled cattle exemplify three genetically diverse cattle populations.

Importantly, across breeds, genes with known cell type-specific functions demonstrated promoter DNA methylation, promoter chromatin accessibility and RNA-seq expression profiles that were most often specific to the corresponding cell type(s) (Fig. 1C). For example, the promoter of the *CD4* gene being specifically unmethylated and open in CD4+ T cells, with the gene’s expression restricted to these cells.

**Fig. 2:**
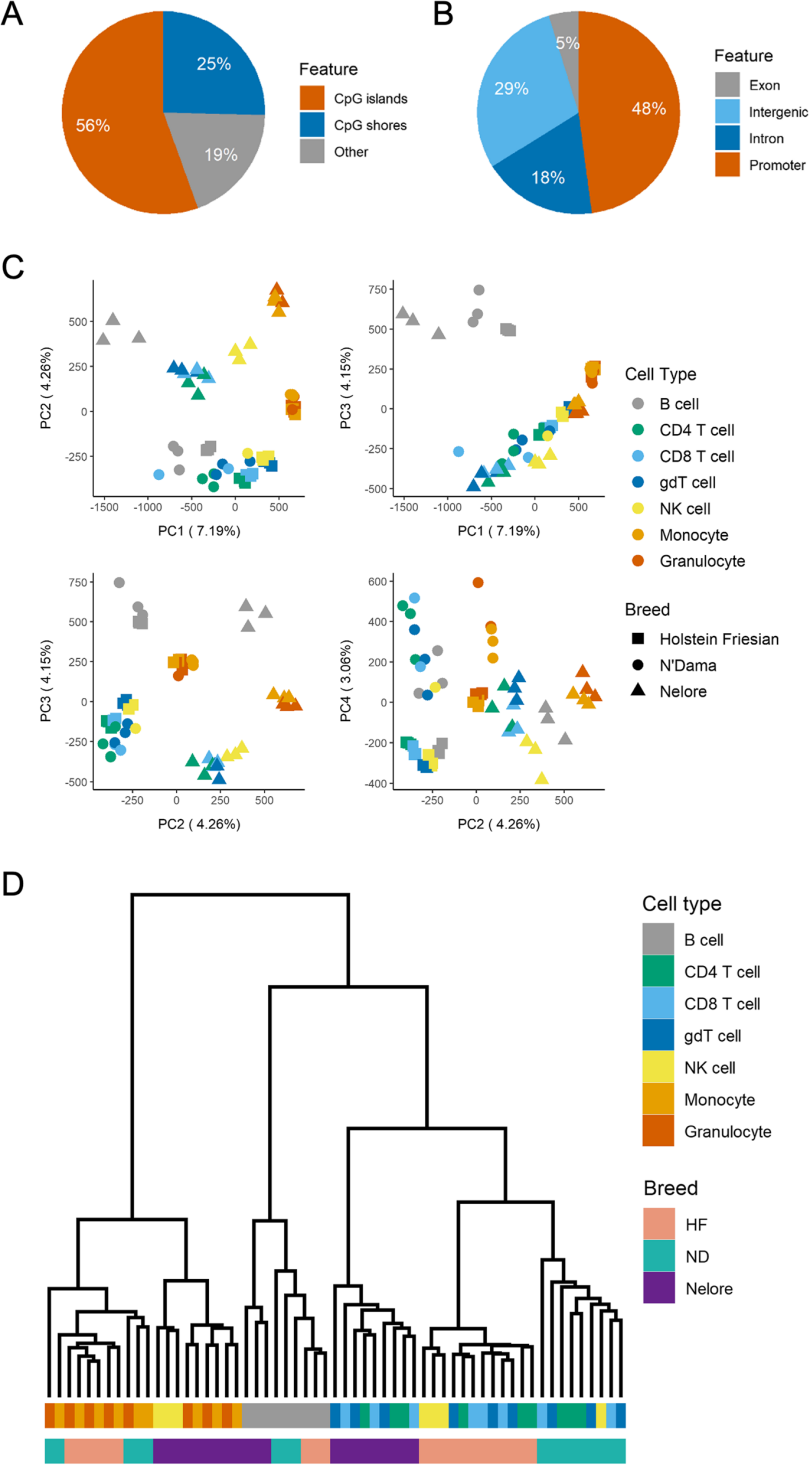
DNA methylation divergence between cell types and cattle lineages. CpG sites were restricted to those covered by at least 10 reads in all samples (9084 sites). **A**, **B** Annotation of CpG sites overlapping defined genomic features. Where CpG sites overlapped multiple genomic features, precedence was given as follows: promoter > exon > intron. **C** PCA of percentage methylation of CpG sites. **D** Unsupervised hierarchical clustering of samples based on their percentage methylation at CpG sites. Samples are clustered based on the Spearman’s rank correlation distance between them for the top 50% of CpG sites displaying the highest standard deviation across all samples (4542 sites). gdT cell denotes γδ T cell

Consistent with expectations, the ATAC-seq data showed clear periodicity in fragment lengths, concordant with the fragments spanning different numbers of nucleosomes (Additional file 1: Fig. S1A). The RRBS data was substantially enriched at CpG islands (CGIs) with 56% of CpG sites covered by at least 10 reads in all samples positioned at CGIs, and a further 25% positioned within CGI shores, defined as 2 kb regions flanking the CGIs (Fig. 2A). In comparison, only around 1% of the reference genome contains CGIs. Consistent with many gene promoters containing CGIs, 48% of CpG sites were positioned at promoter regions (Fig. 2B).

To further validate the data, we checked the concordance between the datasets. In agreement with previous studies [36–39], we confirmed that the accessibility of a promoter was significantly correlated to the expression of the corresponding gene on a global scale (*r*_s_ = 0.53–0.56, *P* < 0.0001). We also observed that a higher proportion of unmethylated CGIs (< 10% methylated) were associated with more highly expressed genes compared to methylated CGIs (> 90% methylated) (Additional file 1: Fig. S1D-E). Furthermore, as expected, we found that CGI methylation was inversely associated with CGI chromatin accessibility (Additional file 1: Fig. S1D-F).

The global distribution of CpG site percentage methylation was bimodal, with an enrichment of unmethylated sites reflecting typically unmethylated CGIs (Additional file 1: Fig. S2B). Interestingly, B cells displayed a higher proportion of intermediately methylated (10–90% methylated) CpG sites relative to the other cell types across all three breeds. Consistent with previous studies, the variation in CGI percentage methylation between cell types was higher at regions distal to the transcription start site (TSS), where higher proportions of intermediately methylated CGIs were also observed (Additional file 1: Fig. S2C) [40–43]. Together, these results suggest these datasets are consistent with a high quality trans-omic representation of the cattle immune regulome.

### Extensive DNA methylation divergence between cattle lineages

Principal component analysis of CpG site percentage methylation showed a clear separation of samples by not only cell type but also cattle subspecies (Fig. 2C). PC2 reflects variation between the two major domestic cattle subspecies: taurine (Holstein Friesian and N’Dama) and indicine (Nelore), and PC1 reflects differences in cell types. Notably, both taurine breeds cluster closely in this PC1 vs PC2 plot, but when plotting PC2 versus PC4, all three breeds cluster separately. This included N’Dama 1 clustering alongside its other breed members (Additional file 1: Fig. S3). Unsupervised hierarchical clustering of samples based on percentage methylation of CpG sites and CGIs showed largely distinct classification of cell types from myeloid and lymphoid lineages, suggesting the highest level of epigenetic divergence among samples to be between these cell lineages (Fig. 2D, Additional file 1: Fig. S2A). There was further separation of the lymphoid branch into two sub-branches that separated the B cell samples from the other lymphocyte samples (Fig. 2D). Within the myeloid, B cell and lymphocyte clusters, samples predominantly separated by breed, such that samples from the same breed were generally more similar than samples from the same cell type. For example, a Holstein Friesian γδ T cell appears to be more similar to a Holstein Friesian CD4 T cell than to an N’Dama or Nelore γδ T cell, based on percentage methylation of CpG sites and CGIs. This suggests that there are key differences between animals from different locations that eclipse differences between some immune cell types. These differences may reflect differences in both breeds and environments, but the clustering together of the two taurine breeds from different continents suggests an elevated divergence between indicine and taurine subspecies.

### Exploring differential methylation between cell types and cattle lineages

**Fig. 3:**
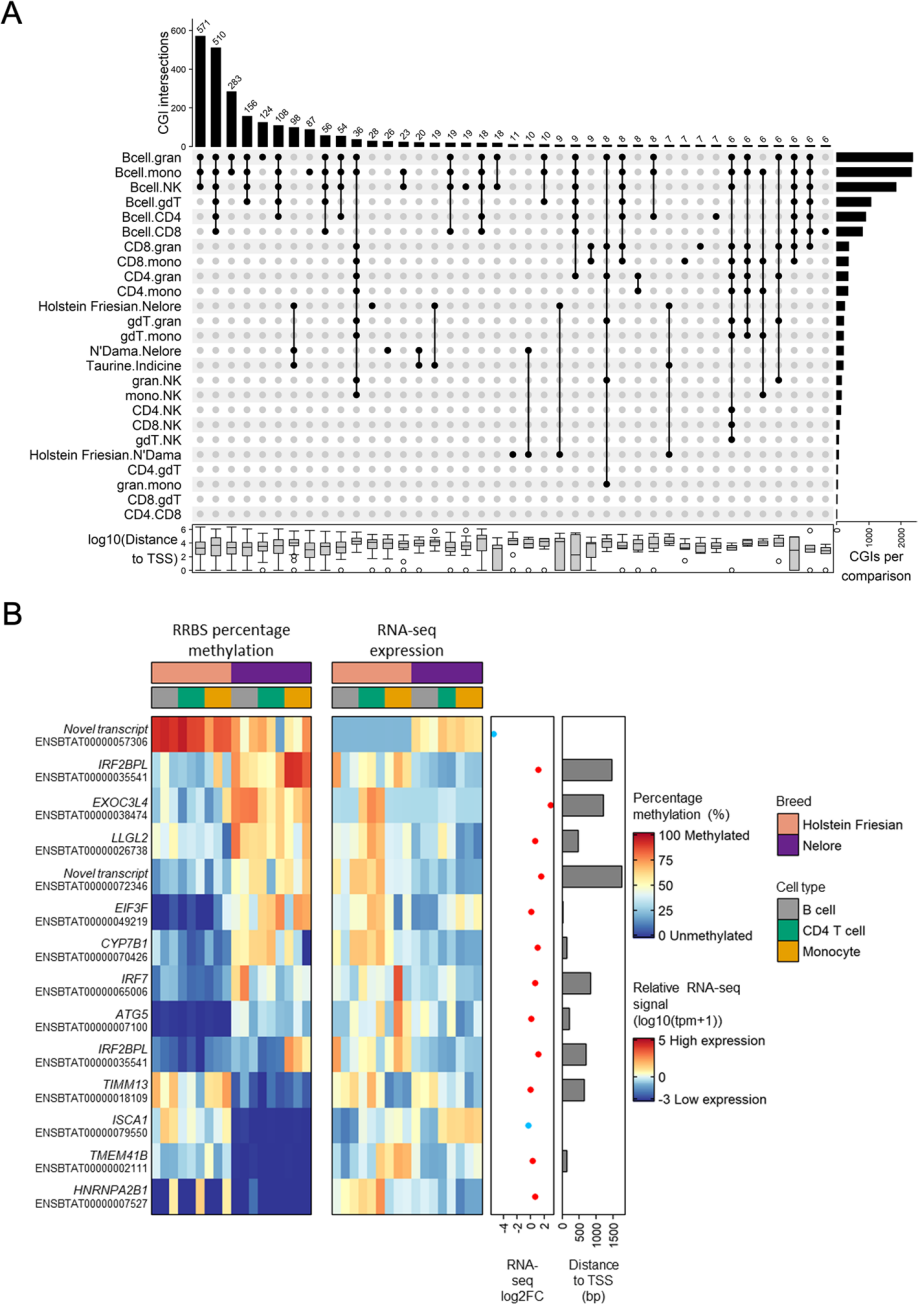
Comparisons of differentially methylated CGIs between cell types, cattle breeds and cattle lineages. **A** Upset plot of differentially methylated CGIs (methylation difference ≥ 25%, *q* value ≥ 0.01) identified in labelled pairwise comparisons. The bars on the right hand side show the number of significantly differentially methylated CGIs in each comparison. The annotated bars along the top show the number of CGIs significantly differentially methylated across the comparisons indicated by black dots. Only sets of comparisons involving at least 6 significantly differentially methylated CGIs are shown. The box plot along the bottom shows the distribution of distances of the CGIs within each combination set to their nearest TSS of a protein-coding gene. The text along the left hand side denotes the groups compared in each pairwise comparison, where ‘.’ separates group 1 and group 2. **B** Heatmap of CGIs differentially methylated between Holstein Friesian and Nelore and/or between taurine (Holstein Friesian and N’Dama) and Nelore. CGIs were restricted to those within 2000 bp of a TSS and where the mean expression of the nearest gene was >10 TPM across all samples. The RNA-seq log2FC is the log2 fold change between the mean Holstein Friesian RNA-seq signal and the mean Nelore RNA-seq signal, where positive log2FC (red points) indicate higher expression in the Holstein Friesian cattle and negative log2FC (blue points) indicate higher expression in the Nelore cattle. Bcell = B cell, CD4 = CD4 T cell, CD8 = CD8 T cell, gdT = γδ T cell, NK = NK cell, mono = monocyte and gran = granulocyte

We compared the CGI methylation levels of samples via pairwise comparisons of cell types, breeds and cattle subspecies. In total, 24,598 CGIs were analysed across the comparisons, of which 3036 CGIs were significantly different in at least one comparison (*q* value ≤ 0.01, percentage methylation difference ≥ 25%). The co-occurrence of differentially methylated CpG islands (DMIs) between pairwise comparisons is visualised in Fig. 3A. Consistent with B cells showing the most distinct profiles in Fig. 2D, the majority (2675) of these DMIs were identified in comparisons of B cells to another cell type. In total, 348 DMIs were identified in at least one of the breed or subspecies comparisons, and 200 of these had a unique methylation profile in the indicine Nelore compared to the two taurine breeds.

To explore this further, we identified the most significantly different CGIs between Holstein Friesian and Nelore where there was matching RNA-seq data. To more accurately associate a CGI with a gene, we restricted CGIs to those within 2 kb of a TSS and where the corresponding gene had an average RNA-seq read count above 10 TPM across samples. This left fourteen CGIs differentially methylated between the animals from these breeds (Fig. 3B). Interestingly, two of these genes (EIF3F and TMEM41B) fall in the vicinity of a genomic region on chromosome 15 (Fisher’s exact test enrichment *P* value = 0.0027) that has previously been significantly linked to survival outcome following *Theileria parva* exposure [44]. The tolerance haplotype at this locus is thought to have arisen in *Bos indicus* cattle, with *Bos taurus* cattle highly susceptible to this pathogen, and these data suggest there may also be elevated epigenetic divergence at this region between the two sub-species. This is also consistent with previous work that has shown that this region is under positive selection between cattle breeds at the genetic level [35].

### Links between genetic and epigenetic divergence between cattle lineages

**Fig. 4:**
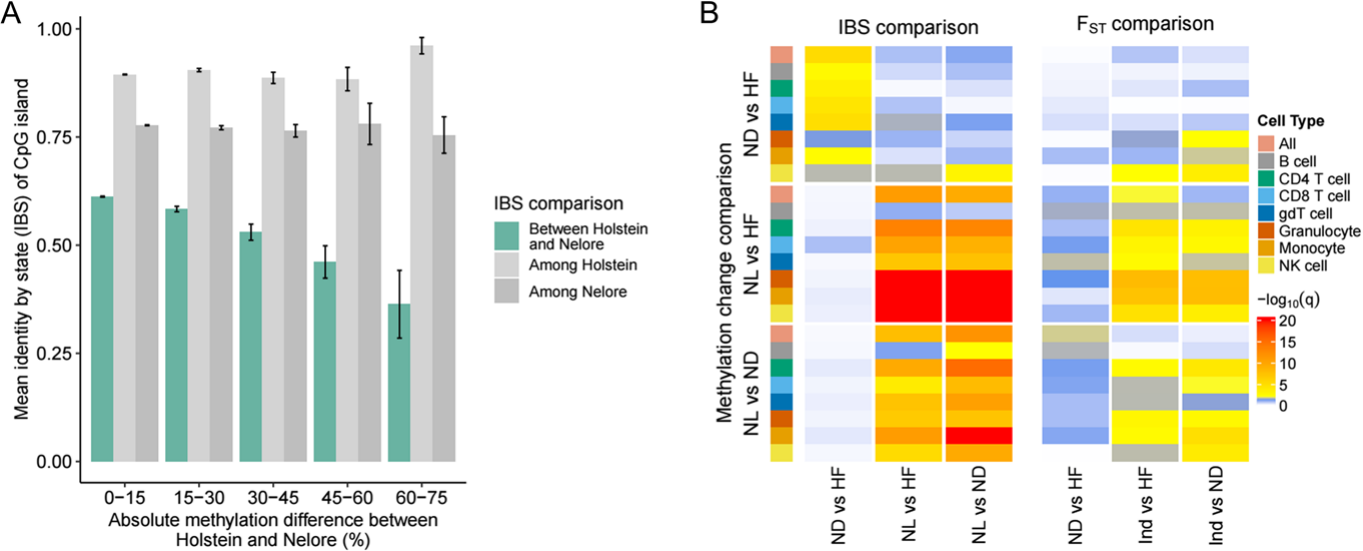
CpG islands of elevated methylation divergence between populations, on average, show elevated genetic divergence. **A** The mean identity by state of CGIs showing different levels of methylation divergence between the Holstein Friesian and Nelore animals when looking across all cell types together. Standard errors of means are shown. CGIs of comparatively elevated methylation divergence generally also show elevated genetic divergence (lower IBS) compared to other CGIs between this pair of populations (shown in green). In contrast methylation divergence between the populations is largely not associated with the IBS scores calculated within the individual populations (shown in grey). **B** Association between IBS (left) or F_ST_ scores (right) and methylation divergence by cell types and population comparison. Each cell indicates the strength of association between the methylation divergence in a particular cell type and population comparison (rows) and the genetic divergence between a pair of populations (columns). Significant associations (corrected FDR *P* < 0.05) are shown in yellow/red, with insignificant results in white/blue. Abbreviations correspond to the following: HF = Holstein Friesian, ND = N’Dama, NL = Nelore, Ind = Bos indicus

To further investigate the relationship between genetic and epigenetic divergence between populations, we calculated identity by state (IBS) scores at each CGI. IBS is a measure of the extent to which alleles at polymorphic sites in a defined region are common between animals. A lower IBS score indicates that the populations share less alleles across the variants in a region, consequently indicating elevated genetic divergence at the locus. As shown in Fig. 4A, those CGIs displaying the greatest methylation divergence between the Nelore and Holstein Friesian animals also, on average, displayed the lowest IBS scores. This is consistent with increased methylation divergence at CGIs reflecting increased genetic divergence at the loci. In contrast, IBS scores calculated among just the Nelore or Holstein Friesian animals separately show little association with the levels of methylation divergence, indicating that regions of elevated genetic divergence within a population are not correlated to methylation divergence between populations. This association between IBS scores and methylation divergence is consistent across cell types and population comparisons (Fig. 4B). In each cell type, methylation divergence between the Nelore and either taurine population shows a significant association with corresponding IBS scores, when controlling for the IBS scores observed within each population and the number of polymorphic sites in the locus. This link between methylation and genetic divergence is not specific to the taurine population being compared to Nelore but reflects sites of general epigenetic divergence between the taurine and indicine lineages. This suggests that the observed methylation divergence is linked to divergence between the lineages more than to any methylation patterns specific to the set of taurine animals or their environments. The sites of elevated epigenetic divergence between the two taurine populations (N’Dama and Holstein Friesian) also show, on average, elevated genetic divergence between the same two populations. However, these sites are distinct to those showing elevated divergence between the taurine and indicine breeds, with no significant association between the sites of elevated epigenetic divergence between the two taurine populations, and the IBS scores between the Nelore and taurine populations (and vice versa). These results indicate the observed epigenetic divergence between populations at least in part mirrors the underlying genetic divergence at the loci. The results were also broadly consistent when calculating IBS after first excluding any variants overlapping a CpG site (Additional file 1: Fig. S4). Consequently, this link between methylation and genetic divergence cannot solely be explained by genetic variants that directly disrupt CpG sites, and therefore their methylation states, between breeds. To further test for links between differentiation at the genetic level and methylation divergence, we also calculated F_ST_ values between cohorts of Holstein Friesian, N’Dama and Nelore cattle (see methods) and compared these scores to the observed methylation differences (Fig. 4B). As with the IBS results, associations were observed between methylation divergence at CGIs between the indicine and taurine breeds and genetic differentiation between the two cattle sub-species.

### Classification of CpG islands into six clusters associated with distinct epigenetic profiles in each breed

**Fig. 5:**
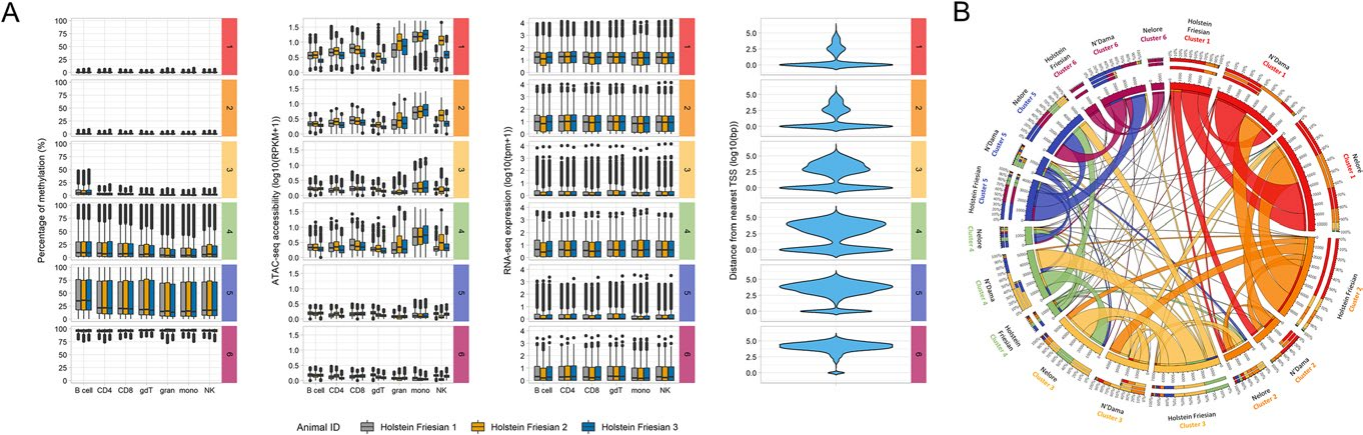
Unsupervised clustering of CGIs identifies distinct chromatin landscapes. **A** CGIs were clustered based on their percentage methylation and ATAC-seq signal (RPKM) in the Holstein Friesian data. Clustering was performed using finite Gaussian mixture modelling (GMM) fitted by the expectation-maximisation (EM) algorithm. Each CGI was only assigned to one gene which had the closest TSS. The RNA-seq expression values for the nearest gene to each CGI are shown as well as the distance of each CGI from the nearest TSS. Clusters are ordered by increasing median percentage methylation and are numbered according to this order. This clustering was repeated for the N’Dama and Nelore data (see Additional file 1: Fig. S5). **B** Circos plot showing the degree of CGI overlap across different clusters between breed pairs. Precedence is given as follows: Holstein Friesian > N’Dama > Nelore. The outermost labels indicate the breed data used in the clustering analysis followed by the cluster number. The outermost bars show the relative overlap of CGIs in each breed that fall within the clusters of the other two breeds. This is also shown in the innermost and middle bars for the Holstein Friesian clusters and Nelore clusters respectively. Specifically for the N’Dama clusters, the innermost bar shows the contribution of CGIs to the Nelore clusters, while the middle bar shows the contribution of CGIs to the Holstein Friesian clusters. The ribbon colours represent different cluster numbers and the ribbon size is equivalent to the proportion of CGIs within a cluster. Bcell = B cell, CD4 = CD4 T cell, CD8 = CD8 T cell, gdT = γδ T cell, NK = NK cell, mono = monocyte and gran = granulocyte

Traditionally DNA methylation has been thought of as binary, with hypomethylation associated with gene activation and hypermethylation repression. However, various studies in humans and mice have suggested this relationship is not so simple, with, for example, intermediate methylation states associated with distinct gene regulatory profiles and pathways [45, 46]. To explore the relationships between patterns of DNA methylation and chromatin accessibility at CGIs in cattle, unsupervised clustering was performed using a Gaussian mixture model (see methods). The model was provided with the percentage methylation and normalised ATAC-seq read counts at corresponding CGIs, where CGIs were restricted to those including at least one CpG site covered by ≥ 5 RRBS reads in all samples of a given breed. Rather than just the two classical sets of hypo- and hypermethylated regions, six distinct clusters were identified for each breed (Fig. 5A and Additional file 1: Fig. S5). For the Holstein Friesian, the first two clusters (clusters 1–2) and the last cluster (cluster 6) correspond to the classical assumptions of CGIs that DNA methylation is inversely correlated with accessibility and gene expression levels (Fig. 5A) [47–49]. Clusters 1–2 corresponded to unmethylated and open CGIs at active promoters (> 80% of CGIs at annotated promoters), while cluster 6 corresponded to methylated and closed CGIs positioned distal to TSSs (76% of CGIs over 5 kb from a TSS) (Fig. 5A, Additional file 1: Fig. S6A). However, clusters 3–5 showed more variation in their percentage methylation between samples, with clusters 4 and 5 containing a high proportion of intermediately methylated CGIs. Intriguingly, cluster 3 included mostly unmethylated CGIs yet the mRNA levels of the associated genes were relatively low. The low methylation status and partial openness of the CGIs in this cluster suggest that they might be associated with genes primed for expression. Unsupervised clustering of CGIs based on their percentage methylation and chromatin accessibility in N’Dama and Nelore revealed broadly similar patterns (Additional file 1: Fig. S5). Overall, this analysis has demonstrated that sets of CGIs display more complex patterns of DNA methylation, chromatin and transcription than the traditional binary picture of active and repressed CGIs.

To compare the classification of CGIs between breeds, CGIs were restricted to those including at least one CpG site covered by ≥ 5 RRBS reads in all samples. The degree of CGI classification overlap between breeds clusters for these 13,810 CGIs is shown in Fig. 5B. In general, CGIs were assigned to the same or nearby cluster number across breeds, suggesting these states are relatively stable across breeds and lineages. However, some links were observed between very different clusters, suggesting a subset of CGIs show substantial divergence in their chromatin and methylation patterns between the cattle groups. For example, 202 CGIs that were categorised into cluster 3 based on their epigenetic profile in Holstein Friesian were categorised into cluster 6 based on their profiles in N’Dama (Additional file 1: Fig. S6B). Gene ontology (GO) term enrichment of the genes nearest these divergent CGIs that were within 2 kb of a TSS revealed enrichment for terms related to DNA binding and transcription factor activity as well as an association with the H3K27me3 regulatory mark at their promoters (Additional file 2: Table S4).

The GO terms enriched within each cluster also revealed extensive overlap between similar clusters of different breeds, but differences between divergent clusters both within and across breeds (Additional file 1: Fig. S6C). Across breeds, genes associated with cluster 1 were generally enriched for housekeeping functions, such as DNA and RNA metabolic processes. This is in agreement with other studies describing that genes that were both highly accessible and highly expressed were enriched for similar processes, such as mRNA processing [38, 50]. In contrast, intermediate clusters are associated with terms such as morphogenesis and cell-cell signalling. The repressed clusters were less enriched with specific GO terms, potentially in part due to largely being distal CGIs that could be less confidently assigned to specific genes.

### Deconvolution of complex cell mixtures based on their DNA methylation profiles

As discussed, EWAS have the potential to disentangle the role of epigenetics and environment on trait variation, but differences in cell type composition between samples can contribute to biased estimates of association and non-reproducible findings. We hypothesised that the reference DNA methylation profiles generated in this study could enable correction of cell type composition between blood samples and thus facilitate EWAS in cattle breeds. As there are over 800 cattle breeds, of particular interest is whether deconvolution profiles obtained from one breed can be used to accurately deconvolute mixed cell samples from another breed, particularly if the breeds are from the different bovine ancestral lineages. To test this, we applied the deconvolution algorithm CIBERSORTx to a filtered set of CpG sites (see methods) to determine the contribution of each individual cell type to six artifical in vitro cellular mixtures and three lysed blood samples for each breed. The in vitro cellular mixtures consisted of two purified cell subsets from Holstein Friesian cattle admixed at defined proportions, and the proportions of each reference cell type in the lysed blood samples were measured using flow cytometry.

**Fig. 6:**
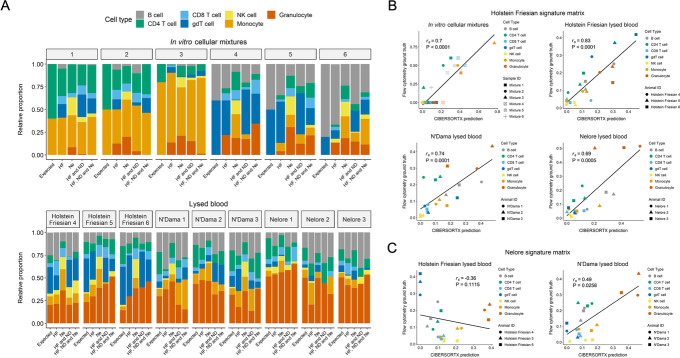
Deconvolution of cellular mixtures based on their DNA methylation profiles. **A** Bar plots of relative proportions of cell subsets in each in vitro mixture and lysed blood sample. For the in vitro cellular mixtures, ‘expected’ refers to the known proportions of FACS-purified cell types used to generate the mixtures. For the lysed blood samples, ‘expected’ refers to the proportions of each cell subset estimated by flow cytometry. The subsequent four bars for each mixture show the proportions of each cell type estimated by CIBERSORTx using reference samples from specified breeds, where HF denotes Holstein Friesian, ND denotes N’Dama and Ne denotes Nelore. Of note, the Holstein Friesian lysed blood samples were collected from different cattle from which the reference profiles were derived, while the N’Dama and Nelore lysed blood samples were collected from the same cattle as the reference profiles. **B**, **C** Scatterplots comparing flow cytometry with CIBERSORTx estimates where only the Holstein Friesian (**B**) or Nelore (**C**) reference samples were used to predict the compositions of the mixtures. Concordance was determined by Spearman's rank correlation (rs) and linear regression (solid line). gdT cell denotes γδ T cell

We validated the performance of CIBERSORTx using the cell type reference profiles for Holstein Friesian and Nelore individually, Holstein Friesian and N’Dama combined and all three breeds combined (Fig. 6A). The N’Dama reference profiles were not used on their own due to a lack of biological replicates for some cell types. We found CIBERSORTx was able to accurately deconvolute the lysed blood samples using reference profiles from any combination of breeds, despite often overestimating the proportion of some cell types that were absent from the in vitro cellular mixtures. One exception was for the prediction of the Holstein Friesian and N’Dama cellular mixtures using the Nelore reference profiles. We found that deconvolution using the Holstein Friesian reference profiles achieved reasonably accurate enumeration of the constituent cell types within samples (*r*_s_ = > 0.69, *P* < = 0.0005; Fig. 6B). In contrast, when using the Nelore reference profiles, the cell type deconvolution of the Holstein Friesian lysed blood samples was negatively correlated with flow cytometry estimates (*r*_s_ = − 0.36, *P* = 0.11; Fig. 6C). This is likely partly a reflection of the substantial cell type composition differences between the breeds, with a higher proportion of T cells and lower proportion of granulocytes in the Holstein Friesian lysed blood samples compared to the Nelore lysed blood samples. The methylation profiles of granulocytes were highly consistent between breeds, and highly distinctive from most of the other the cell types, making them more likely to be predicted correctly by CIBERSORTx.

The accuracy of deconvolution of the N’Dama lysed blood samples when using the reference profiles derived from the Nelore cattle was also lower (*r*_s_ = 0.49, *P* = 0.0258; Fig. 6C) compared to using reference profiles derived from the Holstein Friesian cattle (*r*_s_ = 0.74, *P* = 0.0001; Fig. 6B). These data demonstrate that the reference sample used should ideally be derived from breeds as closely related as possible to the breed from which the cellular mixtures were obtained to achieve the highest deconvolution accuracies. In particular, where a breed from a different lineage is used, the CIBERSORTx predictions may not correlate well with the ground truth proportions. However, when the reference and lysed blood profiles were derived from cattle of the same breed, in this case Holstein Friesian, the predictions were largely accurate (*r*_s_ = 0.83, *P* < 0.0001; Fig. 6B). These immune profiles can therefore serve as effective reference panels for use in EWAS studies to account for cell type composition biases between blood samples.

## Discussion

Here, we report an extensive resource that profiles the methylation and chromatin accessibility landscapes of seven distinct blood immune cell types from both the myeloid and lymphoid immune cell lineages across three genetically divergent cattle breeds representative of the European taurine, African taurine and indicine lineages, with additional RNA-seq data generated for the taurine Holstein Friesian and indicine Nelore cattle. Unsupervised clustering of the percentage methylation separately grouped myeloid cells, B cells and T cells and NK cells, suggesting a strong conservation of the immune system across cattle populations. However, perhaps surprisingly, within these groups, samples often clustered by cattle lineage rather than cell type. For example, the Nelore CD4, CD8 and γδ T cells clustered together as a group, rather than these T cell subsets clustering by breed. This points towards substantial epigenetic divergence between cattle populations, with the divergence patterns observed consistent with the evolutionary distance between the breeds.

It is important to acknowledge that the differences between breeds could be driven by other confounding factors, such as age, environment and differences in farm management strategies. Due to the availability of animals at each of the sampling sites, the animals could only be matched by age within a breed. As a result, each breed was associated with a distinct age range, and since epigenetic patterns are influenced by age and environment, these could have driven some of the observed differences [51]. We did though observe that the extent of methylation divergence at loci was linked to their levels of genetic divergence between populations as measured by both the IBS and F_ST_ metrics. This is broadly consistent with numerous studies in humans that have reported a widespread association between epigenetic and genetic variation [52–55]. These cattle associations were particularly strong when comparing the methylation and genetic divergence between taurine and indicine animals, irrespective of the specific set of taurine animals used in the comparison. This suggests these observed associations are fundamentally linked to differences between these sub-species rather than the specific age or environment of the particular animals selected.

Irrespective of the cause, these results point to high levels of regulatory divergence between cattle groups that have important implications for other studies. For example, large international consortia such as the functional annotation of animal genomes (FAANG) are overwhelmingly focused on breeds of European origin [56], despite only 8% of cattle worldwide being found on the continent [57]. Therefore, despite the clear importance of these resources, they likely poorly represent a large proportion of the global cattle population. The importance of matching resources is exemplified by our analysis of cellular deconvolution using CIBERSORTx. To achieve the highest deconvolution accuracies, the reference samples needed to be derived from breeds as closely related as possible to the breed from which the cellular mixtures were obtained. We therefore anticipate that these reference panels across different lineages will help to better control for potential confounding by cell type and thus support future EWAS studies across breeds.

Characterisation of global percentage methylation revealed a markedly high proportion of intermediately methylated CpG sites in B cells compared to other cell types, particularly monocytes and granulocytes, across the three breeds. This is suggestive of a higher level of inter-cellular heterogeneity in DNA methylation within the B cell population. A previous study by [58] reported a progressive drop in global CG methylation during both human T and B cell differentiation, with a more pronounced change in methylation observed in the latter [58]. Therefore, the intermediate levels of methylation likely reflect a mixed population of B cells at different developmental stages, as expected within peripheral blood [58]. This likely explains the relatively high number of CGIs identified as differentially methylated between B cells and other cell types.

The largest difference in CGI percentage methylation between cell types was found at regions approximately 3–4 kb from the TSS in either direction. At these regions, a higher proportion of CGIs were partially methylated (10–90% methylated) in all cell types compared to CGIs localised to the TSS, which were mostly unmethylated. This suggests that the methylation of distal CGIs was not only highly variable between cell types but also between alleles and/or across individual cells. Additionally, significantly differentially methylated CGIs were mainly located outside of gene promoters, indicating that these regions are important in cell type specificity. These results support other studies in other species that have identified distal regions to more precisely define cell type identity than promoter elements, using ATAC-seq, Chip-seq and BS-seq data [19, 46, 59–62]. Even at the species level, accessibility of distal elements was found to be significantly less conserved between cattle, pig and mouse, than accessibility at proximal elements, such as promoters [22]. The functional impact and mechanisms by which distal CGIs contribute to gene expression remains poorly understood. It has been proposed that they act as previously undetected or alternative gene promoters [63–65] or contribute to the activity of active enhancers [66–68].

Furthermore, unsupervised clustering of CGIs based on their DNA methylation and chromatin accessibility identified six discrete groups for each breed, with each linked to distinct transcriptional states. While the first and last clusters followed the classical association of methylation with nuclease-resistant chromatin and silencing of gene expression, this relationship was largely not maintained in the middle clusters, demonstrating that the epigenetic patterns at CGIs are more complex than a binary on-off transcriptional switch. Comparative analyses showed that the majority of CGIs fell into clusters with similar profiles across breeds, although CGIs of extreme divergence were also observed.

## Conclusions

In summary, we have generated comprehensive datasets providing insights into the interplay of DNA methylation, chromatin accessibility and gene expression across seven immune cell types and three cattle breeds. These data will help to inform the identification of epigenetic patterns and regulatory mechanisms that are conserved, or divergent, across cell types, cattle breeds and species. This will be beneficial for understanding the processes underpinning phenotypic variation across evolutionary lineages, for functionally validating genetic variants associated with a trait, and to contribute to cattle genetic improvement programmes to breed animals with more robust immune systems.

## Methods

### Sample collection and cell isolation

The Holstein Friesian, N’Dama and Nelore cattle were sampled at The Royal (Dick) School of Veterinary Studies at the University of Edinburgh (Edinburgh, UK), ILRI (Nairobi, Kenya) and the Centro Avançado de Pesquisa Tecnológica do Agronegócio de Bovinos de Corte, Sertãozinho (São Paulo, Brazil), respectively. All sampled animals were female, but due to the availability of animals to be sampled at each location, animals could only be matched by age within sites. At the time of sampling, the Holstein Friesian cattle were approximately 10 months old, the N’Dama cattle were approximately 48 months old and the Nelore cattle were between 117 and 141 months old.

The ATAC-seq, RRBS and RNA-seq reference profiles of purified cell subsets were generated from the same nine cattle (three cattle of each breed), and these cattle will subsequently be referred to as Holstein Friesian, N’Dama and Nelore 1, 2 and 3. The methylomes of fifteen cellular mixtures were also generated to assess how accurately their composition could be computed using the reference profiles. Six of the cellular mixtures were generated in vitro by mixing purified cell populations from Holstein Friesian cattle 4 and 7 at defined proportions. The remaining nine cellular mixtures were blood samples collected from Holstein Friesian 4, 5 and 6; N’Dama 1, 2 and 3; and Nelore 1, 2 and 3. Lastly, a pilot comparative analysis of RRBS and Whole Genome Bisulfite Sequencing (WGBS) used cell populations isolated from Holstein Friesian 2, 3, 8 and 9.

Blood was sampled via jugular venepuncture into blood bags or 60 ml syringes containing citrate-phosphate-dextrose solution with adenine. Peripheral blood mononuclear cells (PBMCs) were obtained from blood by density gradient centrifugation: 30 ml blood was overlaid onto 20 ml Ficoll Plaque Plus (GE Healthcare) in a 50-ml centrifuge tube and centrifuged at 800×*g* for 30 min with no brakes. Cells at the interface layer were collected and washed once in phosphate-buffered saline (PBS). To lyse red blood cells, cells were incubated in 5 ml ammonium chloride lysis buffer (0.1X 0.175M Tris pH 7.4 + 0.9X 0.16M Ammonium Chloride) at room temperature for 5 min, followed by centrifugation for 10 min at 300×*g*. Cells were then washed twice and resuspended in PBS.

To enrich for granulocytes, following Ficoll density gradient centrifugation, all layers above the RBC layer were removed by pipetting, leaving approximately 5 ml of solution above the RBC layer to ensure it was not disrupted. To lyse RBCs, 10 ml of the RBC layer was incubated with 40 ml ammonium chloride lysis buffer (0.1X 0.175M Tris pH 7.4 + 0.9X 0.16M Ammonium Chloride) at room temperature for 5–10 min, followed by centrifugation for 10 min at 500×*g.* Cells were then washed three times with PBS.

### Flow cytometry analysis and cell sorting

The antibody panels used for flow cytometry are detailed in Additional file 2: Table S6. Due to the difference in flow cytometers that were accessible in each sampling location, amendments to fluorophores used in the screening and sorting of cells from each breed were required to ensure compatibility with the lasers and filters available. Nevertheless, the markers used to define each population of interest were unchanged and equivalent levels of purity were often achieved (Additional file 1: Fig. S7, Additional file 2: S7).

Antibody dilutions were prepared in FACS buffer (PBS + 0.5% BSA + 2mM EDTA). Following antibody staining, cells were resuspended in FACS Collection Buffer (PBS + 2% BSA + 2 mM EDTA). To sort Holstein Friesian and N’Dama cells, 1 μg/ml DAPI was added to the FACS Collection Buffer. The cell sorter used for the Nelore cells did not have a violet laser; hence, DAPI was not used. Fluorescence-activated cell sorting (FACS sorting) of Holstein Friesian, N’Dama and Nelore cells was performed on a Becton Dickinson FACSAria III Cell Sorter, a Becton Dickinson Influx Cell Sorter and a Becton Dickinson FACSAria II Cell Sorter, respectively. Granulocytes were sorted using FACS based on their forward and side scatter profile. A representative gating strategy used for sorting is shown in Additional file 1: Fig. S7. Purity of sorted cell populations was validated by post-sort flow cytometric analysis of 1000 events. All cells were stained and sorted within 9 h of blood collection and kept on ice between processing steps. Sorting was performed to > 94% purity (Additional file 2: Table S7), and then cells were pelleted at 350×*g* for 5 min and resuspended in an appropriate volume of PBS for downstream processing.

Prior to FACS sorting of NK cells and CD8 T cells, a negative pre-sort using anti-IgG magnetic MACS beads (Miltenyi Biotech) was performed to remove B cells, CD4 T cells, monocytes and γδ T cells and consequently enrich for NK cells and CD8 T cells. For this, up to 10^9^ PBMCs were resuspended in culture media (RPMI 1640 Medium + 10% FBS) and incubated with IL-A58, IL-A12, IL-A24 and GB21A antibodies (2 μg/ml; Additional file 2: Table S6) for 15 min at 4 °C. Following two washes with culture media, cells were resuspended in 10 μl immunomagnetic anti-IgG beads (Miltenyi Biotec) per 1 × 10^7^ cells and incubated for 10 min at 4 °C. Cells were then washed and resuspended in 2 ml culture media. Approximately 2.5 × 10^8^ cells were passed through a 40-μm sterile filter onto each LD column (Miltenyi Biotec), which was placed on quadroMACS magnet and the flow through containing the negatively sorted cells collected. Following antibody staining, MACS-sorted cells were then FACS sorted as previously described.

To isolate adequate amounts of RNA from Holstein Friesian NK cells for sequencing, RNA was pooled across three or four NK cell isolations for each animal. To further enrich for NK cells prior to FACS-sorting, T cells were removed during the MACS pre-sort by the addition of 2 μg/ml MM1A to PBMCs prior to addition of the anti-IgG beads.

Due to a fault with the cell sorter, B cells from N’Dama 2 could not be sorted by FACS; thus, this sample was isolated using MACS positive selection. For this, 2.5 × 10^7^ PBMCs were resuspended in MACS buffer (PBS/0.5% BSA), and cells were incubated with 0.3 mg/ml IL-A58 for 15 min at 4 °C. Cells were washed, resuspended in 25 μl anti-IgG beads (Miltenyi Biotec) and incubated for 10 min at 4 °C. Cells were then washed and resuspended in 500 μl MACS buffer. Cells were passed through a 40-μm sterile filter onto a LS column placed on a quadroMACS magnet (Miltenyi Biotec). After washing the column twice with 500 μl MACS buffer, the column was removed from the magnet, and 1 ml MACS buffer was added to the column. The bound cells were flushed out of the column using a plunger, and their cell purity was assessed by post-sort flow cytometric analysis.

Cells recovered from each sorting session were used to generate paired RRBS and ATAC-seq data. The one exception to this was the Nelore 1 granulocytes, where the cells for RRBS and ATAC-seq were isolated on different dates.

### Spike-in of P815 mouse cells

To check for any global changes in methylation or chromatin, cultured P815 mouse cells were washed with PBS and spiked-in to the FACS-sorted Holstein Friesian cells at a 1:10 ratio, respectively, to generate the single cell type reference ATAC-seq and RRBS data. P815 is a mastocytoma cell line derived by methylcholanthrene treatment of a male DBA/2 mouse [69]. The identity of the cells was not authenticated and cultures were not tested for mycoplasma contamination.

### Generation of artificial in vitro cellular mixtures and screening of lysed whole blood

In vitro cellular mixtures were prepared using FACS-sorted cells from Holstein Friesian cattle at 1:1, 4:1 and 2:3 ratios of monocytes to CD4 T cells and B cells to γδ T cells, yielding a total of six artificial mixtures. To prepare whole blood for flow cytometry, RBCs were lysed using lysis buffer as described for the enrichment of granulocytes. The lysed whole blood samples were screened using the same antibodies as used for FACS, with granulocytes identified based on their forward-side scatter. Flow cytometry analyses were performed on a Becton Dickinson LSRFortessa X-20, a Becton Dickinson FACSCanto and a Becton Dickinson FACSMelody Cell Sorter for Holstein Friesian, N’Dama and Nelore cattle, respectively. The proportions of each cell type were used as the reference values to assess the accuracy in lysed blood deconvolution using CIBERSORTx. However, when calculating the sum of all the cell type proportions within PBMCs, 10–36% of cells were of an unknown cell type or were positive for multiple of the cell surface markers screened within a single antibody panel. As no reference DNA methylation profiles were generated for these cell populations, the fraction of these cells within lysed blood could not be estimated. Therefore, to enable the CIBERSORTx predicted proportions to be compared to the flow cytometry proportions, the proportion of unknown cells and cells positive for multiple markers were distributed across the characterised cell types based on their proportions within lysed blood (Additional file 2: Table S8).

### DNA extraction

DNA was extracted from sorted cells, in vitro artificial mixtures and lysed blood samples using a QIAGEN DNeasy blood and tissue kit with proteinase K and RNase treatment (QIAGEN), following the manufacturer’s recommendations. The concentration of the genomic DNA was determined using a Qubit 3.0 Fluorimeter (Invitrogen). Genomic DNA was checked for degradation and contamination by gel electrophoresis.

### Selection of method to study DNA methylation at base pair resolution

We carried out a pilot comparative analysis of RRBS and WGBS data to determine which method captured the highest proportion of CpG sites at sufficient coverage. For this, genomic DNA from CD4 T cells, monocytes, γδ T cells and CD8 T cells was submitted to Diagenode, Belgium, for their RRBS service, which included library preparation using Diagenode’s Premium RRBS Kit and 50 bp single-end sequencing on an Illumina HiSeq 3000 instrument. Genomic DNA from monocytes was also submitted to BGI, Hong Kong, for their WGBS service, including library construction and 150 bp paired-end sequencing on an Illumina HiSeq 4000 instrument. Briefly, WGBS library preparation included fragmentation of genomic DNA to 100–300 bp using sonication. This was followed by DNA end repair, in addition to 3′-adenine overhangs, and ligation of methylated sequencing adapters. DNA was denatured and bisulfite converted using the ZYMO EZ DNA Methylation-Gold kit. Libraries were then desalted, size selected, PCR amplified and size selected again.

We found that for similar sequencing costs the two approaches covered a similar number of CpG sites with ≥ 10x coverage, but RRBS captured more sites within CGIs and required a smaller amount of DNA (Additional file 1: Fig. S8, Additional file 2: Table S9). Therefore, RRBS was used to generate all subsequent DNA methylation data.

### Reduced representation bisulfite sequencing

DNA from sorted cells, in vitro artificial mixtures and lysed blood samples was submitted to Diagenode for their RRBS service. This service included Qubit sample quantification, DNA quality assessment using the Fragment AnalyzerTM and library preparation using Diagenode’s Premium RRBS Kit. Briefly, sample preparation involved digestion of 100 ng DNA using MspI, followed by end repair and addition of methylated control DNA and unmethylated control DNA. Adapters were ligated to the fragments and AMPure beads were used to remove adapter dimers. Samples were quantified using qPCR and pooled. DNA was bisulfite converted and the optimal number of cycles for the enrichment PCR was determined using qPCR. PCR was then performed to amplify DNA fragments, followed by clean-up using AMPure beads. DNA was quantified using Qubit and the fragment size was monitored on a 2100 Bioanalyser (Agilent). The bisulfite-converted DNA was then sequenced on a HiSeq 3000 instrument using 50 bp single-end sequencing.

### ATAC sequencing

Fifty thousand sorted cells in PBS were pelleted in a 96-well v-bottomed plate by centrifugation at 500×*g* for 2 min at 4 °C, and the supernatant was carefully removed. Cell pellets were lysed in 100 μl cold lysis buffer (10 mM Tris hydrochloride, pH 7.4, 10 mM sodium chloride, 3 mM magnesium chloride, 0.1% IGEPAL CA-630) [70]. Immediately following lysis, nuclei were spun at 500×*g* for 10 min at 4 °C. Supernatant was removed by pipetting, and nuclei were resuspended in 50 μl transposase mixture (25 μl 2x Tagment DNA buffer, 2.5 μl TDE1 Tagment DNA (Illumina) and 22.5 μl nuclease-free water) and disrupted by pipetting. Lysed cells were transferred to 1.5 ml microcentrifuge tubes and incubated for 30 min at 37 °C in an Eppendorf ThermoMixer with agitation at 300 rpm. Transposed DNA was purified using a QIAGEN MinElute Reaction Cleanup Kit according to the manufacturer’s recommendations and eluted in 14 μl of nuclease-free water. Transposed fragments were amplified using v2_Ad1.1 (index i5) and v2_Ad2.1 - v2_Ad2.12 (index i7) primers from [71]. To determine the number of PCR cycles required, qPCR reactions were carried out in duplicate 10 μl reactions using 0.5 μl transposed DNA, 1x NEBNext High-Fidelity PCR Master Mix (NEB), 1.25 μM dual-index PCR primers, 0.5X SYBR Green I (Invitrogen) and 15 μM ROX reference dye (Agilent Technologies). Samples were incubated at 72°C for 5 min, then 98 °C for 30 s, followed by thermal cycling for 30 cycles at 98 °C for 10 s, 63 °C for 30 s and 72 °C for 1 min. The normalised reporter signal was plotted against the cycle number, and the cycle number that corresponded to ¼ of maximum fluorescent intensity was determined. The remaining 12.5 μl undiluted transposed DNA was then amplified by the determined number of PCR cycles in 50 μl reactions using 1X NEBNext High-Fidelity PCR Master Mix and 1.385 μM dual-index PCR primers. Libraries were amplified for a total of 11–18 cycles. Amplified DNA was purified using a QIAGEN MinElute PCR Purification Kit, following the manufacturer’s protocol, and eluted in 20 μl nuclease-free water.

Samples were purified using 1.4x AMPure XP beads (Beckman Coulter) to remove DNA fragments below 150–200 bp and eluted in 50 μl nuclease-free water. Additional size selection was performed to remove large DNA fragments (> 1 kb). For this, 0.5x AMPure beads were added to the sample to bind larger DNA fragment, which were discarded. 0.9x AMPure beads were added to the supernatant and DNA was eluted in 20 μl 0.1x TE buffer. Libraries were quantified using a Qubit 3.0 Fluorimeter (Invitrogen), and the insert size was assessed on a 2200 TapeStation System (Agilent Technologies) using High Sensitivity D1000 ScreenTape and Reagents (Agilent Technologies). Samples were pooled into multiple pools based on barcode compatibility. All Holstein Friesian ATAC-seq libraries were submitted for 75 bp paired-end sequencing on an Illumina HiSeq 4000 instrument to yield at least 96M + 96M reads per sample.

To increase the read coverage of ten of the Holstein Friesian ATAC-seq samples, the samples were pooled and re-sequenced using 50 bp paired-end sequencing on an Illumina NovaSeq 6000 instrument to yield approximately 350M + 350M reads per sample. These re-sequenced samples were the granulocyte samples from each animal, the monocyte and γδ T cell samples from Holstein Friesian 2 and 3, the CD4 T cell samples from Holstein 1 and 3 and the NK cell sample from Holstein Friesian 1. The N’Dama and Nelore pooled libraries were submitted for 50 bp paired-end sequencing on a NovaSeq 6000 instrument to yield approximately 83M + 83M reads and 300M + 300M reads per sample, respectively.

### Transcriptome sequencing

Sorted cells were pelleted at 300*×g* for 5 min and resuspended in 700 μl Tri reagent for every 5 × 10^6^ cells. Samples were transferred to QIAshredders (Qiagen), which were centrifuged at 12,000*×g* for 2 min. RNA was then isolated from the homogenates using a miRNeasy mini kit (Qiagen) with on-column DNase digestion, following the manufacturer’s recommendations. RNA quality was assessed using the 2200 TapeStation System. RNA samples were prepared for sequencing using TruSeq stranded mRNA-seq library preparation by Edinburgh Genomics. Samples were sequenced on an Illumina HiSeq 4000 instrument using 150 bp paired-end sequencing to yield at least 80M + 80M reads per sample.

### Whole genome sequencing

Blood was collected into PAXgene blood DNA tubes (Qiagen) and genomic DNA was isolated using a PAXgene Blood DNA Kit (Qiagen), following the manufacturer’s instructions. DNA concentration was quantified on a Qubit 3.0 Fluorimeter (Invitrogen). The purified DNA was submitted to Edinburgh Genomics for TruSeq nano DNA library preparation and whole genome sequencing (WGS) on an Illumina HiSeqX using 150 bp paired-end sequencing to yield ~15x coverage.

### RRBS read alignment and methylation calling

Quality assessment was carried out using FASTQC [72]. The Trim Galore software [73] was used to trim the 3′ end on all reads using a minimum Phred quality score of 20 and to remove adapter contamination. To remove potential methylation-biased bases from the MspI digestion end-repair reaction, a further 2 bp was trimmed from the 3′ end of adapter-trimmed reads given no trimming was performed based on score quality. As a spike-in of mouse cells had been used for some samples, trimmed reads were mapped to the concatenated bovine ARS-UCD1.2 (GCA_002263795.2; [74]) and mouse GRCm38.p5 (GCA_000001635.7) reference genomes using Bismark [75]. The minimum alignment score function was set at L,0,−0.2. Reads aligning to the cow autosomes, X chromosome and mitochondrial genome were extracted prior to extraction of the methylation calls. After adapter trimming and filtering out of low quality data, between 8 and 27 million RRBS reads per reference sample uniquely mapped to the *Bos taurus* (ARS-UCD1.2) reference genome, resulting in a total of 940 million clean single-end reads across all reference RRBS samples (Additional file 2: Table S1). There were between 13 and 31 million filtered uniquely mapped reads per mixture RRBS sample (Additional file 2: Table S5). Following assessment of the percentage methylation across each possible position in the read, the methylation calls for the first 5 bp from the 5′ end and last 3 bp from the 3′ end of reads were ignored to remove potential methylation bias.

### ATAC-seq read alignment and peak calling

Trim Galore software was used to remove Nextera adapters and read pairs where at least one of the two sequences became shorter than 20 bp and to trim the 3′ end on all reads using a minimum Phred quality score of 20. Sequences were mapped to the concatenated bovine ARS-UCD1.2 (GCA_002263795.2) extended with the Y chromosome from the Btau_5.0.1 assembly [76] and mouse GRCm38.p5 (GCA_000001635.7) reference genomes using Bowtie2 [77]. The alignments settings -D 20 -R 3 -N 0 -L 20 -i S,1,0.50 were applied. The maximum fragment length for valid paired-end alignments was 2000 bp, and at most, 10 distinct, valid alignments were reported for each read using the parameter -k 10. For the Holstein Friesian samples that were sequenced on the HiSeq and NovaSeq sequencers, reads were then merged into a single file. Reads aligned to the cow autosomes and X chromosome were extracted and unmapped reads removed. Mapping of ATAC-seq data resulted in a total of 3.5 billion paired-end bovine reads (64 million on average per sample) (Additional file 2: Table S2). The fragment size distributions of the ATAC-seq libraries were then calculated using Picard [78] and for each sample the insert size counts were normalised by dividing by the total number of counts.

Further filtering of reads was performed simultaneously with peak calling using Genrich (available at https://github.com/jsh58/Genrich). Genrich was run separately for each breed-specific cell type. Genrich was run in ATAC-seq mode, PCR duplicates were removed, and peaks called with a false discovery rate (FDR)-adjusted *P*-value above 0.05 were excluded. In ATAC-seq mode, Genrich analyses 100 bp intervals centred on the transposase cut sites. The distance of the midpoint of each read to the nearest Ensembl-annotated TSSs was then calculated using bedtools [79].

### RNA-seq data analysis

Kallisto [80] was used to index the ARS-UCD1.2 (GCA_002263795.2) reference cDNA sequences and to quantify the abundance of transcripts by pseudoalignment of reads to the reference with 100 bootstraps. Following pseudoalignment, between 39 and 139 million Holstein Friesian and Nelore mRNA-seq reads were mapped to the reference (Additional file 2: Table S3).

### Whole genome sequencing variant calling

Alignment and variant calling was performed for the nine cattle used in this project, alongside a further 289 cattle (see Availability of Data and Materials) (using the same methods as described in [35]). Variants were then filtered using plink 1.90b4 [81]. Firstly, animals with missing variant call frequencies > 0.1 were removed (--mind 0.1). Next, variants with missing call frequencies above 0.05 and variants with a minor allele frequency below 5% were removed (--geno 0.05 --maf 0.05). Lastly, principal components were computed using the --pca parameter for the remaining variants, where the number of components was equal to one less than the number of animals included in the analysis.

### Quantification of ATAC-seq reads and RRBS percentage methylation

The coordinates of CGIs across the bovine genome (ARS-UCD1.2) were calculated using EMBOSS [82] and promoters were defined as 1000 bp upstream and 500 bp downstream of Ensembl-annotated TSSs.

The number of Genrich output ATAC-seq reads that overlapped CGIs and promoters were then counted using bedtools where at least 50% overlap was achieved. The read counts were normalised to reads per kilobase of feature per million reads mapped (RPKM) by first dividing by the region length and then by the total number of Genrich-output reads, divided by 1 million.

For analysis of CpG methylation levels, percentage methylation of individual CpG sites was calculated using the cytosine report files from the Bismark methylation extractor and the Methylkit package in R [83]. CpG sites with high (above 99.9th percentile of coverage in each sample) and low (below 10x coverage) read coverage were excluded. CpG site coverage was normalised using a scaling factor based on median CpG coverage. CpG site percentage methylation was then calculated by dividing the number of reads containing a cytosine at a given CpG site by the total coverage of that CpG site. CGI and promoter percentage methylation were calculated using Methylkit by dividing the number of reads containing a cytosine within a region by the region coverage.

### Calculation of the distance between genomic features

The least genomic distance between two genomic features (CpG site, TSS, CGI, promoter) was calculated using the R package valr. Where a feature was a region, the distance was calculated from the start or end of the region to the nearest upstream or downstream feature.

### Principal component analysis and sample correlation

The ATAC-seq PCA was performed using normalised read counts at CGIs where the sum across all samples was > 50 RPKM and where the counts were scaled across samples using the scale function from base R. The RRBS PCA was conducted using the percentage methylation of CpG sites covered by at least 10 reads in all samples. The RNA-seq PCA was performed using the transcripts per million values for each sample.

Spearman’s rank correlation between samples of each data type was calculated using the same inputs as used for the PCA, and samples were clustered using complete linkage hierarchical clustering. Further hierarchical clustering based on percentage methylation was performed using CpG sites with over 10x coverage in all samples that had a standard deviation across samples within the upper 50% quantile of all sites. Spearman’s rank correlation between samples was then calculated and samples were clustered using the Ward1 method.

### Annotation of CpG sites and islands

The percentage of CpG sites overlapping the promoter (defined as 1000 bp upstream and downstream of the TSS), exon, intron or intergenic regions was calculated using the R package genomation [84]. For CpG sites that overlapped multiple genomic features, precedence was given as follows: promoter > exon > intron. The percentage of CpG sites overlapping CGIs or CGI shores was calculated using Methylkit.

### Differential methylation analysis of CpG islands

Methylkit was used to perform CGI differential methylation analysis using a chi-squared test with correction for overdispersion for the following pairwise comparisons: (i) all samples compared between cattle subspecies, (ii) all cell types compared between each breed and (iii) all breeds compared between each cell type. For the analysis between cattle lineages, the Holstein Friesian and N’Dama samples, both representing breeds of the taurine lineage, were compared to the indicine Nelore samples, where cell type was fitted as a covariate. To compare all cell types between breeds, pairwise comparisons were performed where each breed was compared to the other two breeds, with cell type again fitted as a covariate. Differential methylation analysis was also performed for all breeds between cell types, with breed fitted as a covariate. Following the differential methylation test, a sliding linear model was used to adjust *P* values to *q* values.

An Upset plot was then generated to explore the relationship between pairwise comparisons. For this, a significantly differentially methylated CGI was assigned 1 where the *q* value was ≥ 0.01, and the methylation difference was ≥ 25%; otherwise, a CGI was assigned 0. An UpSet plot showing the top 40 sets containing the highest number of CGIs was created using the R package ComplexHeatmap.

CGIs were then restricted to those that were significantly differentially methylated between Holstein Friesian and Nelore and/or between taurine (Holstein Friesian and N’Dama) and Nelore. CGIs were further restricted to those within 2 kb of a TSS and where the mean expression of the nearest gene was >10 TPM across all samples.

### Linking genetic and epigenetic divergence

Plink version 1.90p was used to calculate IBS scores at each CGI, having excluded all variant sites where more than one animal had an uncalled genotype. IBS scores were calculated both between all pairs of populations, as well as within each of the three populations separately. Only CGIs with at least one CpG site covered by at least five reads were retained, with the small number of islands with a negative IBS score also being excluded. Multiple linear regression was used to test for associations between IBS scores and methylation divergence while controlling for within population IBS scores and the number of variants in the CGI. *P* values were converted to *q* values to account for multiple testing. To test whether variants disrupting CpG sites were solely driving the association between IBS and methylation divergence, we first identified all CpG sites in the genome using the Biostrings R package. The bedtools subtract function was then used to exclude variants intersecting one of these sites and IBS and *q* values recalculated as before.

To calculate F_ST_ scores, we compiled cohorts of Holstein Friesian, N’Dama and Bos indicus whole genome sequence derived genotypes from previous studies. As well as the 9 animals from this study, we used data for 33 Holstein-Friesian and 10 N’Dama animals from [35] and 13 N’Dama from [85] (see Availability of Data and Materials). Due to the comparative limited availability of publicly available Nelore genomes, we used data from 14 indicine animals from [35]. These are shown in purple in Fig. 1B and as can be seen cluster very closely to the three studied Nelore. In total, this gave 36 Holstein Friesian samples, 26 N’Dama samples and 17 indicus samples between which we calculated F_ST_ scores at each variant using vcftools having first excluded variants with more than 20% missing genotypes. As for the IBS analysis, only CGIs with at least one CpG site covered by at least five reads were retained. F_ST_ scores were then averaged across the variants within each CGI, and islands with less than four variants were excluded. Mean F_ST_ scores at the remaining islands were then correlated to their absolute methylation differences for each pair of populations using a Spearman’s rank correlation.

### Correlation between RNA-seq gene expression, ATAC-seq chromatin accessibility and RRBS percentage methylation

For Holstein Friesian and Nelore separately, the ATAC-seq RPKM values and the RNA-seq TPM values were averaged across samples at each promoter and transcript, respectively. Non-coding transcripts were removed from the analysis as they were not profiled using mRNA-seq. Spearman’s rank correlation between log_10_-transformed values was then calculated.

To compare between RNA-seq gene expression and percentage methylation, the nearest annotated TSS to each CGI was identified. To ensure transcripts were not associated with multiple CGIs, only the CGIs nearest the transcript were retained in the analysis, but no minimum distance threshold was applied. CGIs associated with non-coding transcripts were removed from the analysis. For each breed, the percentage methylation of each CGI and the TPM values of each transcript were averaged across samples. The CGIs were then split into ten bins based on their percentage methylation and the RNA-seq values for each bin were plotted. For the same CGIs, ATAC-seq RPKM values at CGIs were also averaged across samples and plotted against bins of corresponding CGI percentage methylation.

### Unsupervised clustering of CpG islands based on their methylation and chromatin accessibility profiles

The following steps were performed separately for each breed. Firstly, the percentage methylation and ATAC-seq RPKM values at corresponding CGIs were combined into a single matrix, where CGIs were required to contain at least one CpG site covered by ≥ 5 RRBS reads. CGIs were then restricted to those covered in all samples, and any CGIs with ≥ 50 ATAC-seq RPKM were removed, as they were generally uncorrelated to percentage methylation. The percentage methylation and ATAC-seq RPKM values were scaled within a sample across CGIs. The CGIs were then clustered using the R package mclust 5.4.5, which uses finite Gaussian mixture modelling [86]. Based on the Bayesian information criterion scores for all the available models, the VVV model was used with six components. The RNA-seq TPM values of the corresponding transcripts were then appended to the corresponding CGIs. Non-coding transcripts and associated CGIs were removed from the analysis.

To compare the CGI classification between breeds, CGIs were restricted to those containing at least one CpG site covered by ≥ 5 RRBS reads and ≤ 50 ATAC-seq RPKM in every sample across breeds. The number of CGIs falling within each combination of clusters across breed pairs was then calculated, where the clusters were ordered by increasing median percentage methylation within a breed. The sharing of CGIs between different breed clusters was then visualised as a circos plot using the Circos application [87]. GO term enrichment analysis was performed for CGIs within 2 kb of a TSS that were classified in Holstein Friesian cluster 3 and N'Dama cluster 6. Since a high proportion of these genes had human orthologs, functional annotation analyses were performed using the GENE2FUNC function of the web-based platform FUMA v1.3.6a [88]. FUMA was run using default settings with Benjamini–Hochberg multiple testing correction.

GO term enrichment analysis was also performed on the nearest genes to the CGIs within each breed-specific cluster. For this, transcripts were restricted to those where the TSS was situated inside or within ± 10 bp of their associated CGI, as these could be more reliably associated with the given transcript than a TSS further from a CGI. Enrichment analyses were performed for each cluster using FUMA, with genes across all clusters for a given breed as background.

### Individual CpG site differential methylation, CIBERSORTx application and signature matrix generation

Differentially methylated CpG sites were identified in pairwise comparisons of unmixed, individual cell types using Methylkit, which uses a chi-square test with correction for overdispersion. Differential methylation statistics were calculated using (i) the Holstein Friesian reference samples, (ii) the Nelore reference samples, (iii) Holstein Friesian and N’Dama reference samples and (iv) the reference samples from the three cattle breeds. Differential methylation was not performed on the N’Dama samples alone, since for some cell types only one or two samples were collected.

CpG sites were selected where the differential methylation *q* value was ≤ 0.1 across any cell type comparison, with no restriction on the percentage methylation difference. A matrix containing the percentage methylation at these CpG sites in each sample of the individual cell types was used to generate a reference file for upload to CIBERSORTx [89]. The percentage methylation values of the cellular mixtures at the same CpG sites were extracted to generate a corresponding mixture file. Only CpG sites that were covered by all the samples were retained in the reference and mixture files, which were then uploaded to CIBERSORTx. The CIBERSORTx signature matrix consisting of ‘barcode’ CpG sites was generated from the reference file using default CIBERSORTx parameters (300–500 barcode CpG sites were considered for each cell type). To impute the fraction of cell types within the cellular mixtures, CIBERSORTx was run using 1000 permutations. Deconvolution accuracy was determined by Spearman’s rank correlation between CIBERSORTx predicted and expected proportions. For the lysed blood samples, the expected proportions were measured using flow cytometry, and for the artificial mixtures, the expected proportions were the proportions at which the two cell types were admixed.

## Supplementary Information

**Additional file 1: Fig S1. **Relationship between percentage methylation, chromatin accessibility, and gene expression.** Fig S2. **Global characteristics of DNA percentage methylation.** Fig S3. **The position of N’Dama 1 when clustering by epigenetic state.** Fig S4. **The impact of excluding polymorphic CpG sites when associating genetic and epigenetic divergence.** Fig S5. **Unsupervised clustering of CGIs based on their DNA methylation and chromatin accessibility profiles.** Fig S6. **Functional characterisation of CGI clusters displaying distinct chromatin profiles.** Fig S7. **Sorting strategy for blood cells.** Fig S8. **Comparison of CpG site coverage between RRBS and WGBS.

**Additional file 2: **Table S1. Reference RRBS mapping summary.** Table S2. **ATAC-seq mapping summary.** Table S3. **RNA-seq mapping summary.** Table S4. **Divergent CpG island GO enrichment.** Table S5. **Mixture RRBS mapping summary.** Table S6. **Antibody panels.** Table S7. **Cell sorting purities.** Table S8. **Screening of lysed blood.** Table S9. **RRBS vs WGBS mapping summary.

**Additional file 3: Table S10. **ENA sample accessions of animals used in this study.** Table S11. **Accessions of WGS data used in PCA.** Table S12. **Accessions of WGS data used to calculate FST scores.

**Additional file 4. **Review history.

## Data Availability

The datasets generated for this manuscript are publicly available at The European Nucleotide Archive (https://www.ebi.ac.uk/ena/browser/home) under the accession PRJEB36894 [90]. The cattle IDs used in this manuscript were changed from the IDs used for the uploaded dataset. The corresponding cattle IDs and accession numbers are shown in Additional file 3: Table S10. Whole genome sequencing data of additional cattle used in the genotype PCA and F_ST_ score analyses are available at the ENA under primary accession numbers listed in Additional file 3: Table S11 and Table S12 [35, 44, 91–101] [102–117]. No custom scripts and software was used other than those mentioned in the “Methods” section.
