# Supplementary material for: Profiling the immune epigenome across global cattle breeds
Source: Genome Biol. 2023 May 22;24:127. doi: 10.1186/s13059-023-02964-3 (PMC10204299; doi:10.1186/s13059-023-02964-3)
Supplement: Supplementary file 1 — Additional file 1: Fig S1. Relationship between percentage methylation, chromatin accessibility, and gene expression. Fig S2. Global characteristics of DNA percentage methylation. Fig S3. The position of N’Dama 1 when clustering by epigenetic state. Fig S4. The impact of excluding polymorphic CpG sites when associating genetic and epigenetic divergence. Fig S5. Unsupervised clustering of CGIs based on their DNA methylation and chromatin accessibility profiles. Fig S6. Functional characterisation of CGI clusters displaying distinct chromatin profiles. Fig S7. Sorting strategy for blood cells. Fig S8. Comparison of CpG site coverage between RRBS and WGBS. [file 13059_2023_2964_MOESM1_ESM.docx]

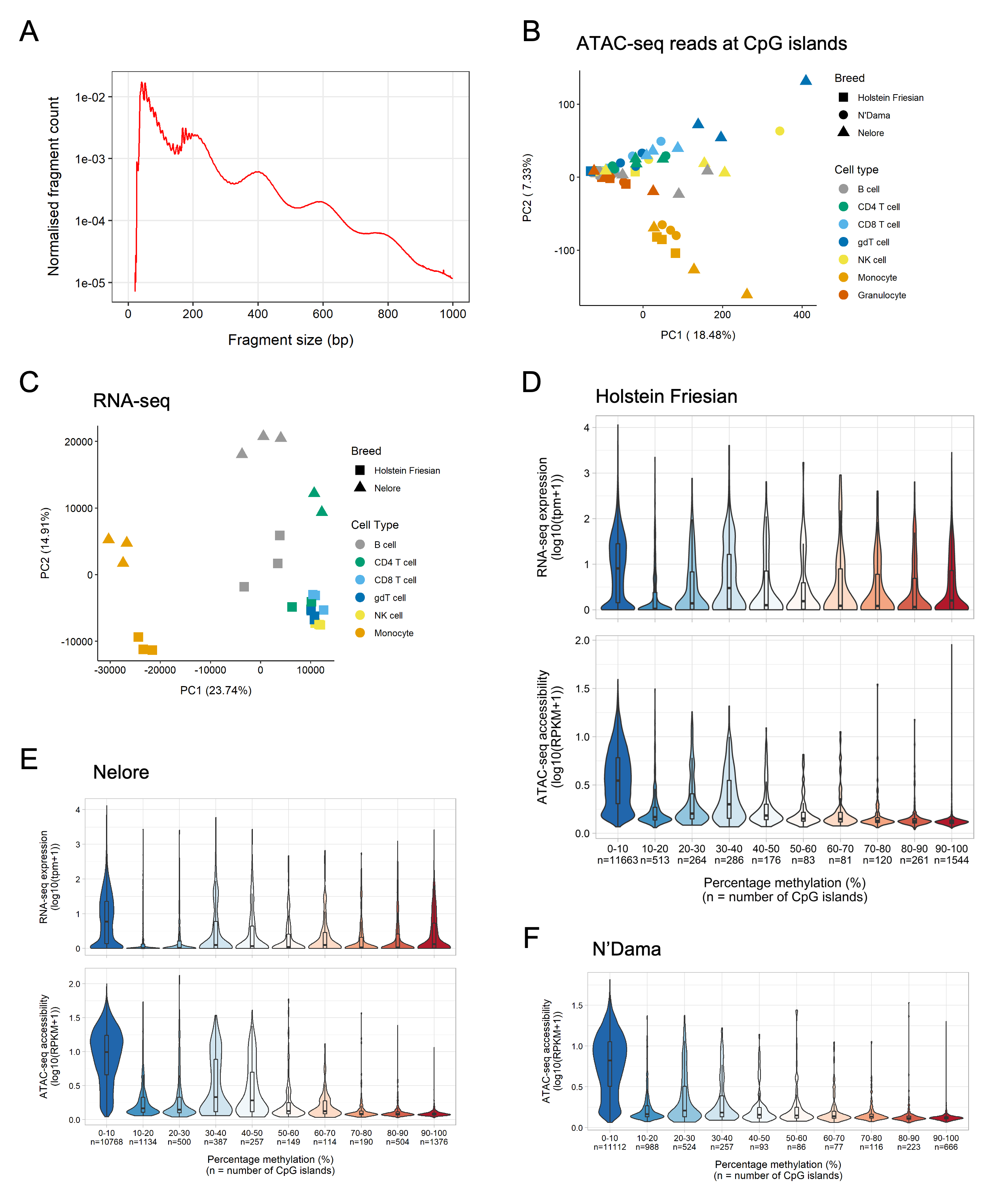


**Fig S1.** **Relationship between percentage methylation, chromatin accessibility, and gene expression.** (A) Histogram of the mean ATAC-seq DNA fragment sizes across all samples. Fragment counts were normalised by dividing by the total number of fragment counts for each sample. (B) PCA of ATAC-seq data using CGIs with a total > 50 RPKM across all samples. (C) PCA of RNA-seq data using TPM for all mRNA-encoding genes. (D-F) CGIs containing at least one CpG site covered by ≥ 5 reads were grouped into ten bins based on their average percentage methylation across samples for each breed. For Holstein Friesian (D) and Nelore (E) CGI percentage methylation was compared to the RNA-seq expression of the nearest gene (top) and the ATAC-seq chromatin accessibility at the same CGI (bottom). For N’Dama, the percentage methylation of CGIs was compared to their corresponding ATAC-seq chromatin accessibility only.


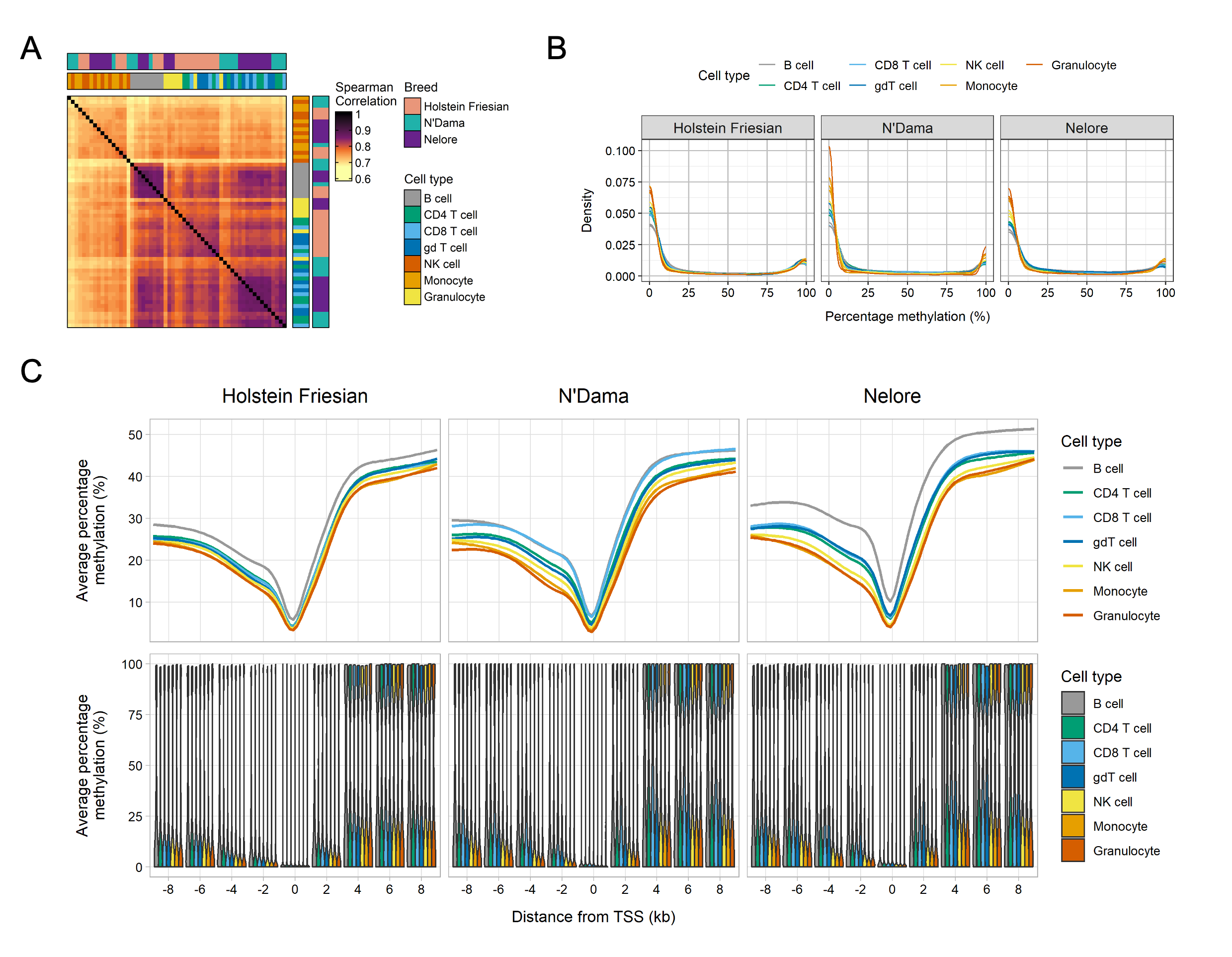


**Fig S2.** **Global characteristics of DNA percentage methylation.** (A) Unsupervised hierarchical clustering of CGI percentage methylation data from all samples. Values shown are Spearman’s rank correlation coefficients. Complete linkage hierarchical clustering was performed using CGI percentage methylation data for all CGIs containing at least one CpG site covered by a minimum of 5 reads in all samples. (B) Density plots of genome-wide DNA methylation levels of CpG sites covered by at least 10 reads across all samples (9,084 sites in total). (C) Comparison of DNA methylation levels across cell types for each breed at CGIs relative to their distance to the nearest TSS. Upper panel shows the mean percentage methylation at CGIs across cell types for each breed. Lower panel shows the distribution of percentage methylation at CGIs within 2 kb bins, up to 9 kb from the TSS. CGIs were restricted to those containing a minimum of one CpG site covered by a least 5 reads in all samples. gdT cell denotes γδ T cell.


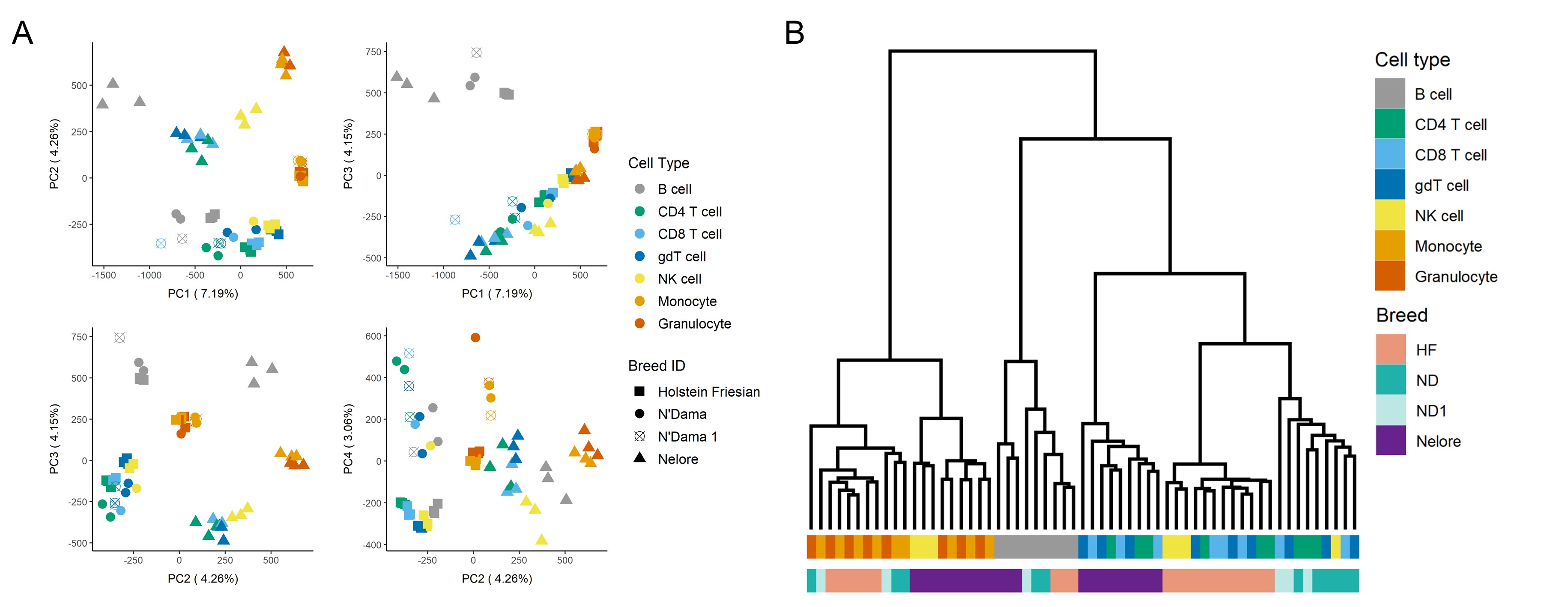


**Fig S3.** **The position of N’Dama 1 when clustering by epigenetic state.** The same results as shown in Figure 2C and D but highlighting the location of N’Dama 1 which was a comparative outlier in the genetics PCA (Figure 1B).


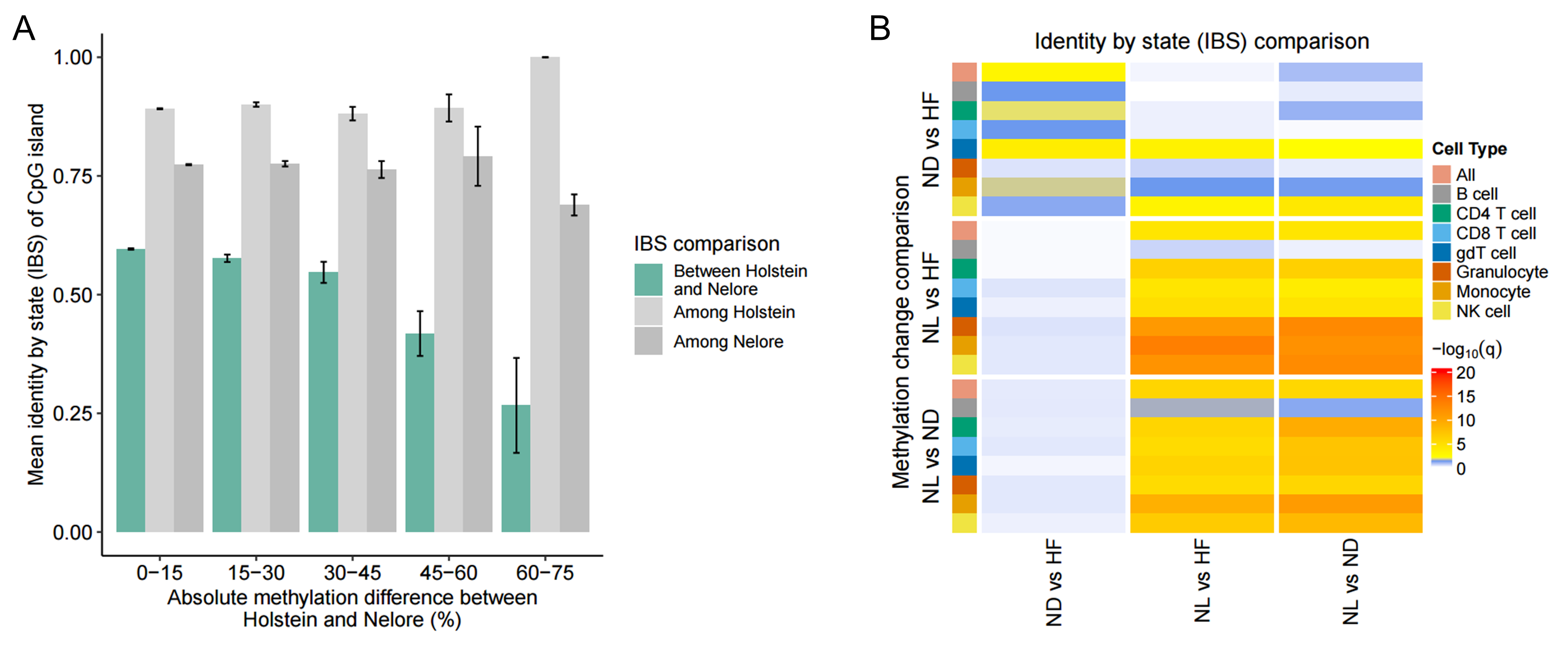


**Fig S4.** **The impact of excluding polymorphic CpG sites when associating genetic and epigenetic divergence.** CGIs of elevated methylation divergence between populations, on average, show elevated genetic divergence even when excluding variants overlapping CpG sites. This Figure is the same as the IBS analysis shown in Figure 4 with the exception that variants overlapping CpG sites were first excluded. The general results match those in the main figure highlighting that the associations observed were not purely due to variants disrupting CpG sites and their methylation states. (A) The mean identity by state of CGIs showing different levels of methylation divergence between the Holstein-Friesian and Nelore animals when looking across all cell types together having excluded variants at CpG sites. Standard errors of means are shown. Sites of elevated methylation divergence generally also show elevated genetic divergence (lower IBS) between this pair of populations (shown in green). In contrast methylation divergence between the populations is largely not associated with the IBS scores calculated within the individual populations (shown in grey). (B) Association between IBS and methylation divergence by cell types and population comparison. Each cell indicates the strength of association between the methylation divergence in a particular cell type and population comparison (rows), and the genetic divergence between a pair of populations (columns). Significant associations (corrected FDR p<0.05) are shown in yellow/red, with insignificant results in white/blue.


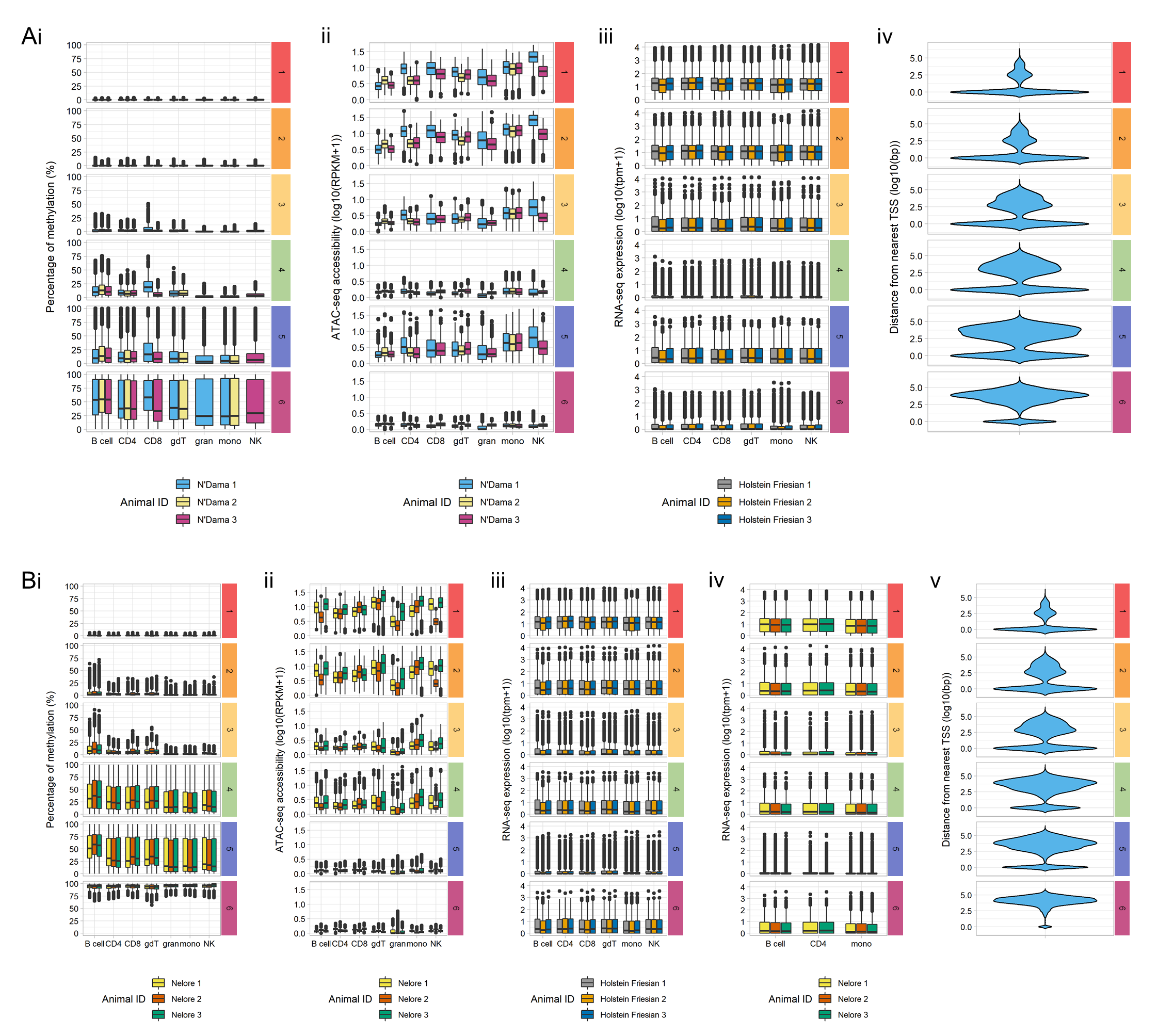


**Fig S5.** **Unsupervised clustering of CGIs based on their DNA methylation and chromatin accessibility profiles.** CGIs were clustered based on their percentage methylation and ATAC-seq signal (RPKM) in the N’Dama (A) and Nelore (B) data. Clustering was performed using finite Gaussian mixture modelling (GMM) fitted by the expectation-maximization (EM) algorithm. The Holstein Friesian RNA-seq expression values for the nearest gene to each CGI are shown for each cluster due to a lack of available RNA-seq data for N’Dama and Nelore. Clusters are ordered by increasing median percentage methylation and are numbered according to this order. The same clustering method was performed using the Holstein Friesian data (Figure 5).


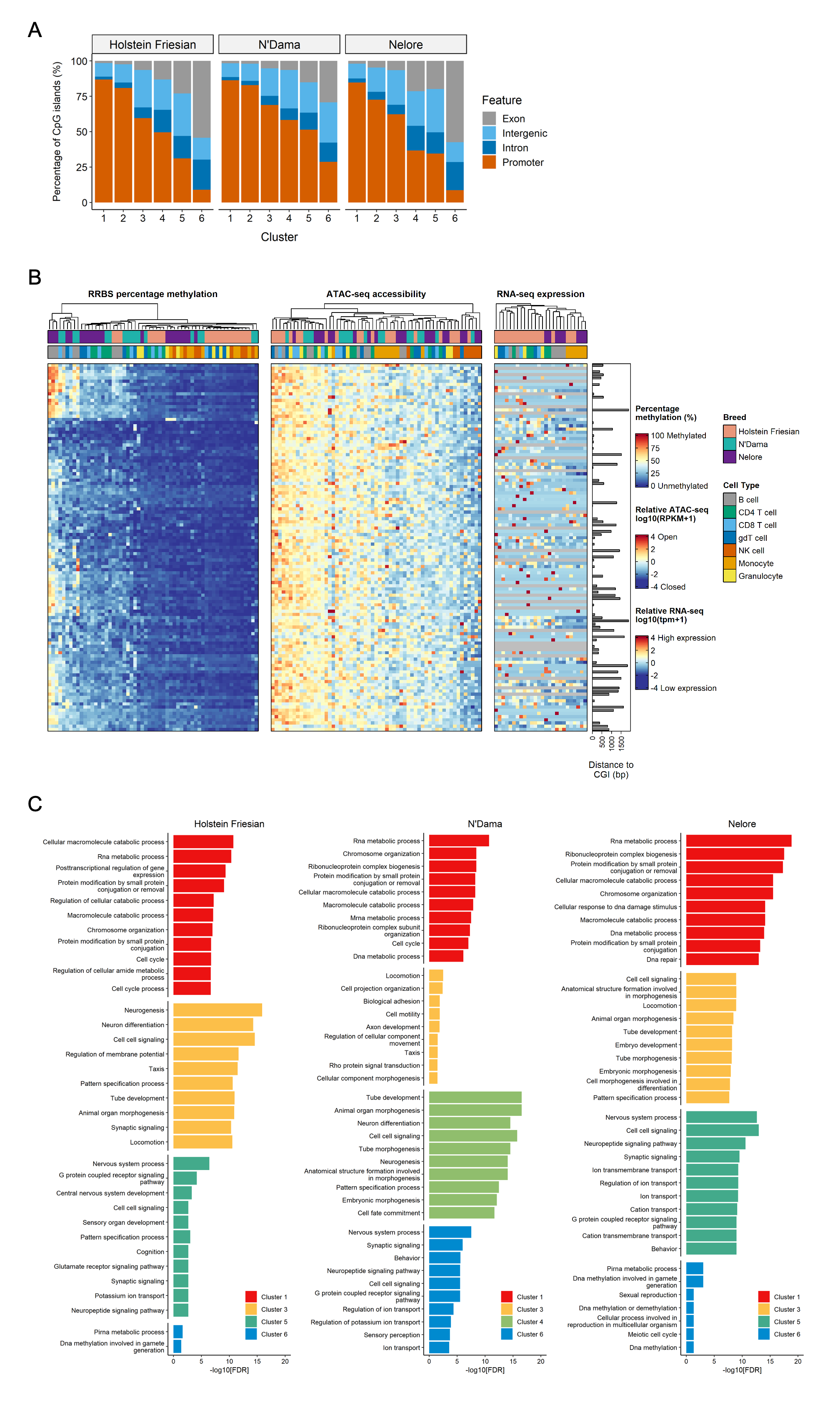


**Fig S6.** **Functional characterisation of CGI clusters displaying distinct chromatin profiles.** The clusters correspond to those shown in Figure 5 and Supplementary Figure 5. (A) Annotation of CGIs within each cluster overlapping promoters, introns, exons, or intergenic regions. For CGIs that overlap multiple genomic features, precedence was given as follows: promoter > exon > intron. (B) Heatmap of CGIs categorised in cluster 3 based on their percentage methylation and ATAC-seq signal (RPKM) in the Holstein Friesian data and cluster 6 based on their corresponding N’Dama data. CGIs were restricted to those within 2000 bp of a TSS. Grey lines in the RNA-seq expression heatmap indicate transcripts with no RNA-seq expression values. (C) Enriched biological process GO terms analysed using FUMA. GO term enrichment was performed using genes nearest each CGI, where the gene’s TSS overlapped or was within 10 bp of the associated CGI, as these CGIs could be more reliably associated with a gene than those distal to the TSS.


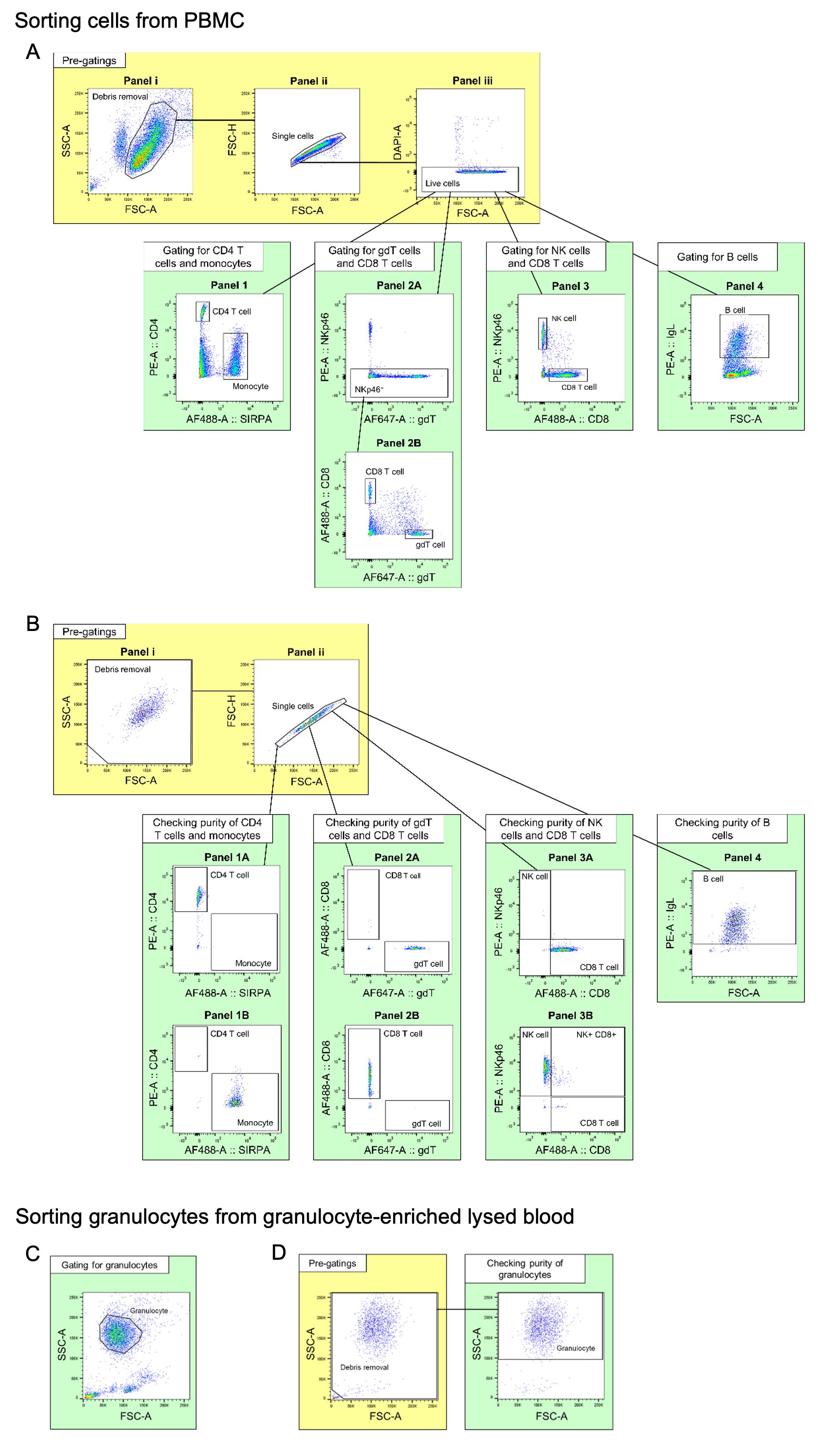


**Fig S7.** **Sorting strategy for blood cells.** (A) Representative examples of the gating strategies used to sort CD4 T cells, monocytes, γδ T cells, CD8 T cells, NK cells and B cells from bovine PBMC. (B) Examples of the gating strategies used to assess the purity of sorted cell populations. Post-sort analyses represent a minimum of 1,000 events. The x and y axes correspond to the intensity of the cell marker staining, where :: separates the fluorophore used and the cell surface marker for a given cell type. SIRPA denotes SIRPα and gdT denotes the γδ TCR. Representative examples of the gating strategy used to (C) sort granulocytes and (B) assess the purity of sorted cell populations.


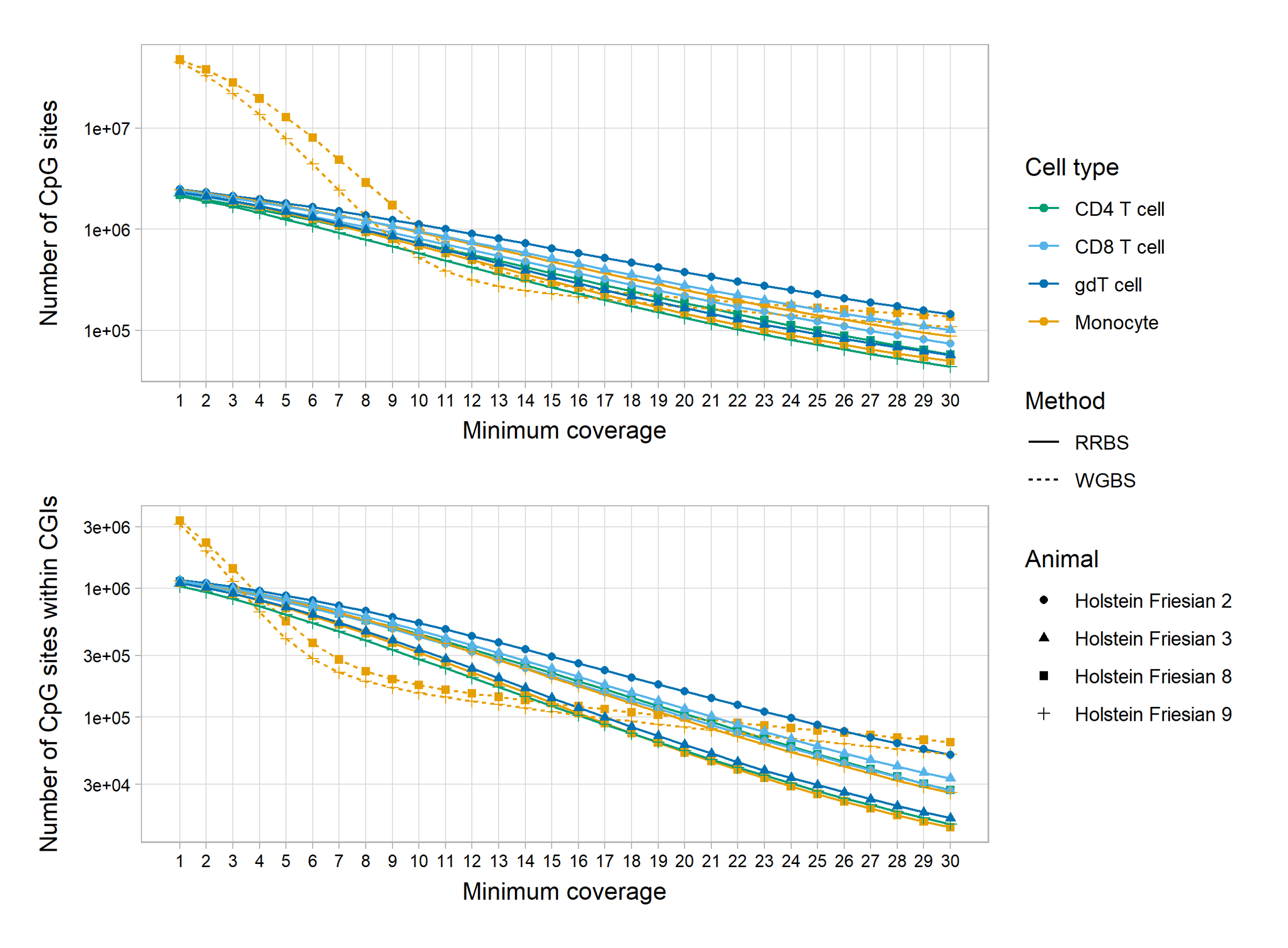


**Fig S8.** **Comparison of CpG site coverage between RRBS and WGBS.** (A) The total number of CpG sites and (B) number of CpG sites within CGIs at given minimum coverage thresholds.
