# Supplementary material for: Profiling the immune epigenome across global cattle breeds
Source: Genome Biol. 2023 May 22;24:127. doi: 10.1186/s13059-023-02964-3 (PMC10204299; doi:10.1186/s13059-023-02964-3)
Supplement: Supplementary file 4 — Additional file 4. Review history. [file 13059_2023_2964_MOESM4_ESM.docx]

Review History

**First round of review**

**Reviewer 1**

**Were you able to assess all statistics in the manuscript, including the appropriateness of statistical tests used?**

Yes, and I have assessed the statistics in my report.

**Comments to author:**

The manuscript by Jessica Powell and co-authors describes a comprehensive study of DNA methylation, chromatin accessibility and RNA expression profiles of the immune system across three distinct cattle breeds. To this end, the authors targeted seven key blood immune cell types and analysed over 150 libraries at base-pair resolution. The resource population was 3 cattle of each of Holstein Friesian, N'Dama and Nelore breeds and breed diversity was verified based on a PCA that included an additional 294 cattle from 56 breeds (or is it 13? …see minor comments).

I believe this work represents a monumental piece of research and a significant advance in the identification of epigenetic patterns and regulatory mechanisms of the immune system. The extensive multi-omics resource has the potential to assist numerous studies. An important practical application is to inform the selection of cattle with more robust immune systems.

I only have two concerns that hopefully the authors can address with further explanation and/or analyses.

The first one is about the sampled animals. In addition of whether three samples from each breed is enough, I'm concerned about the different ages across breeds (Holstein ~ 1 year, N'Dama ~ 4 years and Nelore ~ 10 years) and how this can impact the methylation profile known to be affected by and increase with age. In cattle terms, those ages are equivalent to adolescence, mature and old. Similarly, the fact that the three breeds have been bred and raised in three vastly different environments makes me wonder if some of the epigenetic differences observed are due to breed or due to environment. Put another way: would a Holstein bred in Brazil present epigenetic signatures similar to those of Nelore cattle?

The second concern refers to the way the exploration of differential methylation between cell types and cattle lineages and the clustering of CpG islands is presented and discussed. The authors report a total of 24,598 CGI and 3,036 DMIs (line 213 onwards). The authors list "interesting" genes in these DMIs (eg. EIF3F and TMEM41B) and enriched GO terms (eg. cell-cell signalling perhaps being the most related to immune function?). I'm surprised no attempt is made to confirm if any of these DMI fall in regions reported to harbour signatures of selection for cattle domestication and adaptation to tropical environment. There is a wealth of literature on that subject.

Minor comments:

Line 100: The work by Alexandre et al. (2021) is not with European taurine cattle, but indicine.

Line 158: I believe the work by Dutta et al. (2020) describes 13 cattle breeds, not 56.

Line 916: Spell out ECF as East Coast Fever.

**Reviewer 2**

**Were you able to assess all statistics in the manuscript, including the appropriateness of statistical tests used?**

No, I do not feel adequately qualified to assess the statistics.

**Comments to author:**

This manuscript reports DNA methylation data, ATACseq data, and limited RNAseq data from isolated blood cell types from three cattle breeds, and determines breed-specific regulatory elements and their relationships within and across breeds and cell types. The integrated analysis identifies a number of interesting results. Of highest interest is the Gaussian model derived classification of identified regulatory elements, as well as the deconvolution analysis across breeds. The authors make the important point that current reference data is mostly from the European taurine breed and the data in this manuscript demonstrates additional populations should be measured as there is significant variation among taurine and indicine breeds. The fact is that most of the epigenetics work, especially in the FAANG project, has been focused on taurine breeds. However, the conclusion drawn that "irrespective of the cause, these results point to high levels of regulatory divergence between cattle groups" (in the second paragraph of the discussion) is perhaps too strong, given that a) they correctly indicate that environmental effects (both age stratification and physical environment) are both significant inputs to differential methylation differential expression and b) that they're clearly confounded with these breed samples. Perhaps the authors could map breed-specific SNPs against the breed-divergent regions to demonstrate their conclusion that genetics is causing more (most?) of these differences.

Results presented in Figure 4 are especially interesting; the classification of CGIs was informative and the data was nicely illustrated. In 4B, it is unclear what is depicted by the different concentric rings on the outside of the figure- this is not in the legend, and the color scheme is unclear as it doesn't seem to match previous color schemes such as for cell types. I also would recommend that the authors make more prominent their correct limiting of gene-CGI or gene-ATAC-seq peak relationships so that a gene is not assigned to more than one of these features. I could only find this in the Methods; at least indicate this in the text or Figure legend as the authors discuss differences between proximal and distal elements relative to a gene, as I immediately wondered whether a gene could be assigned to a proximal and a distal element.

Line 227-235. The authors state that the methylation level of CGIs "were most often inversely associated with expression of the associated gene". I find this relationship pretty noisy in the Figure, and while there seems to be instances where this inverse relationship is clear, there are some instances where this is not; for example, one of the genes they specifically mention (TMEM41B) has very low methylation as well as low expression in Nelore cattle. It would be more convincing to run the actual correlations, as well as show these examples.

From supplementary Figure 1 it appears that the CD 4 T cell population and the CD8T cell population both would be expected to contain double positive T cells. While this cell population is low in cattle, I'm not sure that this has been tested across the breeds used here (measured only in Taurine breeds? Only in Holstein Freisan?). If not, could the authors comment on the possible implications of differential DP T cells?

Line 160. The authors describe that one of the N'Dama cattle is likely to be a cross between an N'Dama and a European taurine animal; however there was no follow up of this to indicate whether this animal clearly was different for many of the breed differences they describe in the rest of the manuscript-- was this checked or followed up on?

There are several statements in Results that perhaps should be relegated to Discussion; i.e., lines 211-212.

Line 270. The example of 202 CGIs which have different classifications between two breeds is very interesting. However the GO enrichment discussed was apparently across all CGI; it would be quite interesting to look at the GO enrichment of those 202 CGIs (the genes near them) to understand the function of these genes.

Supplemental figure 3B. It is unclear why the myeloid cells do not cluster in the data for ATACseq reads in CPG islands, as they do at all CPG sites. Can the authors discuss possible reasons for this, especially since (line 200) they describe myeloid lineages being distinct for CPG methylation. Therefore it would seem that you would expect this to be true for accessible CPG islands.

**Authors Response**

**Point-by-point responses to the reviewers’ comments:**

Reviewer #1:

The manuscript by Jessica Powell and co-authors describes a comprehensive study of DNA methylation, chromatin accessibility and RNA expression profiles of the immune system across three distinct cattle breeds. To this end, the authors targeted seven key blood immune cell types and analysed over 150 libraries at base-pair resolution. The resource population was 3 cattle of each of Holstein Friesian, N'Dama and Nelore breeds and breed diversity was verified based on a PCA that included an additional 294 cattle from 56 breeds (or is it 13? …see minor comments).

I believe this work represents a monumental piece of research and a significant advance in the identification of epigenetic patterns and regulatory mechanisms of the immune system. The extensive multi-omics resource has the potential to assist numerous studies. An important practical application is to inform the selection of cattle with more robust immune systems.

I only have two concerns that hopefully the authors can address with further explanation and/or analyses.

The first one is about the sampled animals. In addition of whether three samples from each breed is enough, I'm concerned about the different ages across breeds (Holstein ~ 1 year, N'Dama ~ 4 years and Nelore ~ 10 years) and how this can impact the methylation profile known to be affected by and increase with age. In cattle terms, those ages are equivalent to adolescence, mature and old. Similarly, the fact that the three breeds have been bred and raised in three vastly different environments makes me wonder if some of the epigenetic differences observed are due to breed or due to environment. Put another way: would a Holstein bred in Brazil present epigenetic signatures similar to those of Nelore cattle?

***Reply: In response to the comments by both reviewers, in order to try and disentangle the influence of genetics and age/environment on differential epigenetic profiles we have now added additional analyses investigating the potential link between genetic and epigenetic divergence observed between the breeds. These analyses illustrate that the methylation divergence between breeds is indeed linked to underlying genetic divergence. CpG islands that show the greatest methylation change between breeds also, on average, show elevated genetic divergence. For example, the identity by state (IBS) of CpG islands showing the least methylation divergence between Nelore and Holstein-Friesian animals is approximately 70% higher than that of islands showing the greatest epigenetic change (see new Figure 4A). These links are observed irrespective of the comparisons made between the Bos indicus Nelore animals and either of the Bos taurus breeds (see Figure 4B), indicating it is inherently associated with differences between the two cattle sub-lineages, rather than the specific animals collected and their environments. Although environmental and age factors will undoubtedly also play a role in shaping methylation differences, these results illustrate that the extensive genetic divergence between breeds, and Bos indicus and Bos taurus breeds in particular, likely contributes to the observed epigenetic divergence in this analysis, above and beyond any environmental factors. We now discuss this at lines 234 to 269 and 378 to 386.***

The second concern refers to the way the exploration of differential methylation between cell types and cattle lineages and the clustering of CpG islands is presented and discussed. The authors report a total of 24,598 CGI and 3,036 DMIs (line 213 onwards). The authors list "interesting" genes in these DMIs (eg. EIF3F and TMEM41B) and enriched GO terms (eg. cell-cell signalling perhaps being the most related to immune function?). I'm surprised no attempt is made to confirm if any of these DMI fall in regions reported to harbour signatures of selection for cattle domestication and adaptation to tropical environment. There is a wealth of literature on that subject.

***Reply: As well as the IBS analysis above, in response to the reviewer’s comment we have now added an additional analysis of the link between F_ST_ scores and methylation divergence. F_ST_ measures genetic differentiation between pairs of populations, with regions of high F_ST_ often assumed to indicate regions of potential selection. We observe that CpG islands of elevated methylation divergence between the Bos taurus and Bos indicus animals also, on average, show higher F_ST_ scores. As with the IBS analysis this is consistent with the areas of highest genetic differentiation between the two cattle sub-lineages showing elevated methylation divergence. Unlike in the IBS analysis, we did not observe any link between F_ST ­_scores between the two taurine breeds and their methylation divergence. It is possible that this is due to the relative strength of selection between the breeds and/or the shorter time since divergence. However, these results do indicate that methylation divergence between the sub-species is linked to a widely used statistic of population differentiation. We have now added this analysis at lines 264 to 269 and 378 to 386 and Figure 4B.***

Minor comments:

Line 100: The work by Alexandre et al. (2021) is not with European taurine cattle, but indicine.

***Reply: Apologies for the confusion. By saying predominately European taurine, we did not mean to suggest all of the studies were done in European taurine animals, and had included this reference as an example of a study not from European animals. We have revised this sentence to try and make it clearer that most, but not all, studies were done in European animals at line 99.***

Line 158: I believe the work by Dutta et al. (2020) describes 13 cattle breeds, not 56.

***Reply: Apologies, the reviewer is correct - 56 was the number of breeds in the larger set of 427 cattle samples pre-filtering, but the final filtered set of 294 samples spanned 13 breeds. We have now corrected this at line 158.***

Line 916: Spell out ECF as East Coast Fever.

***Reply: This is now done at line 1032.***

Reviewer #2:

This manuscript reports DNA methylation data, ATACseq data, and limited RNAseq data from isolated blood cell types from three cattle breeds, and determines breed-specific regulatory elements and their relationships within and across breeds and cell types. The integrated analysis identifies a number of interesting results. Of highest interest is the Gaussian model derived classification of identified regulatory elements, as well as the deconvolution analysis across breeds. The authors make the important point that current reference data is mostly from the European taurine breed and the data in this manuscript demonstrates additional populations should be measured as there is significant variation among taurine and indicine breeds. The fact is that most of the epigenetics work, especially in the FAANG project, has been focused on taurine breeds. However, the conclusion drawn that "irrespective of the cause, these results point to high levels of regulatory divergence between cattle groups" (in the second paragraph of the discussion) is perhaps too strong, given that a) they correctly indicate that environmental effects (both age stratification and physical environment) are both significant inputs to differential methylation differential expression and b) that they're clearly confounded with these breed samples. Perhaps the authors could map breed-specific SNPs against the breed-divergent regions to demonstrate their conclusion that genetics is causing more (most?) of these differences.

***Reply: In response to comments by both reviewers we have now added analyses comparing the extent of methylation divergence to local genetic differentiation. See responses to reviewer 1 above for more details, but in summary we observe that methylation divergence is indeed linked to local levels of genetic change between the populations, and therefore likely not simply environmental/age related, but partly driven by local genetic divergence. This signal is particularly evident when looking across the two cattle sub-species, Bos taurus and Bos indicus. See lines 234 to 269 and 378 to 386 and new Figure 4 for more details.***

Results presented in Figure 4 are especially interesting; the classification of CGIs was informative and the data was nicely illustrated. In 4B, it is unclear what is depicted by the different concentric rings on the outside of the figure- this is not in the legend, and the color scheme is unclear as it doesn't seem to match previous color schemes such as for cell types.

***Reply: Thanks to the reviewer for this suggestion, we have now colour coded the labels in what is now Figure 5A to match the colours in what is now Figure 5B and expanded the legend to better describe the rings (see lines 531 to 536).***

I also would recommend that the authors make more prominent their correct limiting of gene-CGI or gene-ATAC-seq peak relationships so that a gene is not assigned to more than one of these features. I could only find this in the Methods; at least indicate this in the text or Figure legend as the authors discuss differences between proximal and distal elements relative to a gene, as I immediately wondered whether a gene could be assigned to a proximal and a distal element.

***Reply: We have now added a sentence to the legend of what is now Figure 5 to clarify how each CGI was only assigned to one (i.e. the closest) gene*** ***(see lines 524 to 525).***

Line 227-235. The authors state that the methylation level of CGIs "were most often inversely associated with expression of the associated gene". I find this relationship pretty noisy in the Figure, and while there seems to be instances where this inverse relationship is clear, there are some instances where this is not; for example, one of the genes they specifically mention (TMEM41B) has very low methylation as well as low expression in Nelore cattle. It would be more convincing to run the actual correlations, as well as show these examples.

***Reply: In Supplementary Figures 1D and E we showed the genome-wide relationship between CGI methylation level and the expression levels of the nearest gene. Consistent with previous knowledge unmethylated CGIs are, on average, linked to higher expression levels. But the relationship between methylation and expression is not simple. As we showed in Figure 5 there are different patterns of relationships between methylation, open chromatin and gene expression. So we did not mean to imply here that we expect a consistent, simple relationship between methylation and expression across all genes, and have accordingly removed this sentence from line 226 to avoid confusion.***

From supplementary Figure 1 it appears that the CD 4 T cell population and the CD8T cell population both would be expected to contain double positive T cells. While this cell population is low in cattle, I'm not sure that this has been tested across the breeds used here (measured only in Taurine breeds? Only in Holstein Freisan?). If not, could the authors comment on the possible implications of differential DP T cells?

***Reply: This is an interesting point. We have actually previously measured the number of double positive cells across multiple individual Bos indicus and Bos taurus cattle (of two breeds from each lineage), and found in those cattle that the percentage of double positive cells was 1.43% and 1.16%, respectively (see Makau et al. Vet Immunol Immunopathol. 2020 Dec; 230: 110126). Therefore, this evidence suggests that these numbers are similar across both lineages, and that the number of double positive CD4 and CD8 cells is low in both. Consistent with this, considering the gene expression profiles of CD4 and CD8 in the RNA-seq data in Figure 1C, there also is little evidence of CD8 being expressed in the CD4 T cell population in either the Holstein-Friesian or Nelore samples. Likewise, there is little if any expression of CD4 in the CD8 T cells. So any presence of double positive T cells seems to be limited. We have now added a statement regarding this at lines 480 - 482.***

Line 160. The authors describe that one of the N'Dama cattle is likely to be a cross between an N'Dama and a European taurine animal; however there was no follow up of this to indicate whether this animal clearly was different for many of the breed differences they describe in the rest of the manuscript-- was this checked or followed up on?

***Reply: We have marked up this N’Dama on the percentage methylation PCA plot and dendrogram and found that it clusters closely with the other two N’Dama cattle (see lines 197-198 and Supplementary Figure 3). Therefore, in subsequent analyses this animal was grouped with the other N’Dama cattle.***

There are several statements in Results that perhaps should be relegated to Discussion; i.e., lines 211-212.

***Reply: We have now removed this sentence as this point is already covered in the discussion.***

Line 270. The example of 202 CGIs which have different classifications between two breeds is very interesting. However the GO enrichment discussed was apparently across all CGI; it would be quite interesting to look at the GO enrichment of those 202 CGIs (the genes near them) to understand the function of these genes.

***Reply: We have now performed GO term enrichment using the genes within 2kb of one of these 202 CGIs. The background list of genes included those within 2 kb of a CGI that were assigned to a cluster across all three breeds. We found enrichment for GO terms related to DNA binding and transcription factor activity, suggesting that these CGIs are associated with genes that have a regulatory function, and also observed enrichment for possession of the trimethylated H3K27 (H3K27me3) mark in the promoter of these genes. We have now added this at lines 304 to 309 and as Supplementary Table 4.***

Supplemental figure 3B. It is unclear why the myeloid cells do not cluster in the data for ATACseq reads in CPG islands, as they do at all CPG sites. Can the authors discuss possible reasons for this, especially since (line 200) they describe myeloid lineages being distinct for CPG methylation. Therefore it would seem that you would expect this to be true for accessible CPG islands.

***Reply: It is true that the distinction is less pronounced, but in the ATAC-seq data the myeloid cells are separated from the lymphoid cells on PC2 of what is now Supplementary Figure 1B. In that figure the myeloid cells are below 0 and the other cells generally above 0 on this axis. We think for various reasons you would not necessarily expect to see a perfect concordance between the chromatin and methylation data, including due to the imperfect association we demonstrate between these factors in, for example, Figure 5A. For example, polycomb repressive complexes and other repressive factors can bind to unmethylated DNA but still be associated with inaccessibility to the underlying DNA and transcriptional silencing (e.g. see Li H et al. Polycomb-like proteins link the PRC2 complex to CpG islands. Nature. 2017 Sep 14;549(7671):287-291).***

**Second round of review**

**Reviewer 1**

Thank your for the thorough analyses illustrating that the methylation divergence between breeds is indeed linked to underlying genetic divergence, ie. CpG islands that show the greatest methylation change between breeds also, on average, show elevated genetic divergence. Figure 4 and Suppl Fig. 4 are quite convincing.

Minor issue:

Line 252: "This suggest it reflects"? Please fix.

**Reviewer 2**

Acceptable responses and revisions to concerns.

However, I searched for the data at ENA, there is no data for PRJEB36894 submitted although the manuscript says the data is publicly available. This must be rectified BEFORE acceptance, and is why I rated the manuscript as "minor revision". Furthermore, the data descriptions should be checked for clarity in description of the individual datasets for future metadata use, a crucial and important component of the value of this work.

**Authors Response**

**Point-by-point responses to the reviewers’ comments:**

**Reviewer #1**

Thank your for the thorough analyses illustrating that the methylation divergence between breeds is indeed linked to underlying genetic divergence, ie. CpG islands that show the greatest methylation change between breeds also, on average, show elevated genetic divergence. Figure 4 and Suppl Fig. 4 are quite convincing.

Minor issue:

Line 252: "This suggest it reflects"? Please fix.

*Reply: Updated. Line now reads: “This suggests that the observed methylation divergence is linked to divergence between the lineages more than to any methylation patterns specific to the set of taurine animals or their environments.”*

**Reviewer #2**

Acceptable responses and revisions to concerns.

However, I searched for the data at ENA, there is no data for PRJEB36894 submitted although the manuscript says the data is publicly available. This must be rectified BEFORE acceptance, and is why I rated the manuscript as "minor revision". Furthermore, the data descriptions should be checked for clarity in description of the individual datasets for future metadata use, a crucial and important component of the value of this work.

*Reply: This is now public (see https://www.ebi.ac.uk/ena/browser/view/PRJEB36894) with appropriate metadata describing the data.*
